# Revolutionizing diabetes care with innovative decision-making using cubic intuitionistic fuzzy Schweizer and Sklar power aggregation operators

**DOI:** 10.1016/j.heliyon.2024.e33075

**Published:** 2024-06-19

**Authors:** Bai Chunsong, Usman Khalid, Muhammad Ahsan Binyamin, Jawad Ali

**Affiliations:** a School of Finance and Mathematics, Huainan Normal University, Huainan 232038, China; b Department of Mathematics, Government College University Faisalabad 38000, Pakistan; c Department of Mathematics, Quaid-i-Azam University, Islamabad 45320, Pakistan

**Keywords:** Cubic fuzzy set, Cubic intuitionistic fuzzy set, Power aggregation operators, Schweizer and Sklar (SS) operations, MCDM

## Abstract

The cubic intuitionistic fuzzy set is an expansion of the cubic fuzzy set that displays massive information to demonstrate interval-valued intuitionistic fuzzy sets and intuitionistic fuzzy sets. This increment informs limitations essential in existing frameworks, primarily focusing on the significance of embracing our access for more accurate decisions in compound and unresolved structures. The Schweizer and Sklar (SS) operations are engaged in promoting strong aggregation operators for cubic intuitionistic fuzzy sets through this research. Operators such as cubic intuitionistic fuzzy Schweizer and Sklar power weighted average (CIFSSPWA) and cubic intuitionistic fuzzy Schweizer and Sklar power weighted geometric (CIFSSPWG) are offered that enhance the workability of data aggregation within the cubic intuitionistic fuzzy (CIF) environment when compared to surviving methods. The proposed operators may assist in patient treatment and handling by upgrading decision-making in medical sectors like diabetes care. Moreover, to determine the stability and reliance of the outcomes, sensitivity and comparison studies are richly absorbed by this approach.

## 1 Introduction

In the modern era, it's getting much more complicated to select the optimal choice from numerous possibilities only due to the growing challenges in the world of decision-making. Multi-criteria decision-making (MCDM) is always of great significance in the economic, engineering, education, and medical sectors. MCDM is an approach for determining the most appropriate course of study among any finite array of alternatives based on a sample of attributes by employing adequate means. A variety of techniques, like interval and exact approaches are rapidly used by the experts making decision-making even harder. The Fuzzy Set (FS) theory was introduced by Zadeh [1] in 1965. This has gained an immense approach and proven to be a very useful technique for handling uncertain data. Over the years, FS theory has been used by many researchers in different real-world situations like Financial forecasting [2], Insurance assessment [3], Engineering systems [4], [5], and Education system [6]. In other ways, the main concern of FS theory is the degree of membership of every item ranging from zero to one. It directs to the fact that the opposite of a membership degree is a non-membership degree. However, this method has a drawback. It often falls short of delivering exact results. FS theory was extended by Atanassov [7] in 1986 with the introduction of Intuitionistic Fuzzy Sets (IFS), which consists of membership and non-membership degrees. In IFSs, the sum of the degrees of membership and non-membership always lies from 0 to 1. Hence, IFSs may provide more detailed and adaptable facts when confronting refers to hesitation. IFSs are therefore considered to be the ideal choice given the complexities of everyday situations and the need for managing the inherent characteristics of several physical procedures. Atanassov [8] introduced Interval-Valued Intuitionistic Fuzzy Sets (IVIFS), an advanced build-up of IFS wherein membership levels are denoted by ranges of fuzzy numbers later.

Decision-making is becoming enormously dominant in fields that comprise of pattern recognition, medical diagnosis, and clustering analysis as a result of progressions in economics and social evolution. By now, researchers have been using intuitionistic fuzzy sets (IFSs) and interval-valued IFSs more frequently in these areas. In order to point out different sorts of intuitionistic fuzzy numbers (IFNs), Garg [9], [10] arose interactive aggregation operators. For IVIFNs, Garg [4] introduced geometric aggregation operators and later on, Liu and Wang [11] suggested ordered weighted aggregation operators for DM troubles. By the application of triangular IFNs, Mahmood et al. [12] suggested hybrid aggregation operators to label DM difficulties. Besides, applying the information from IFNs and IVIFNs, researchers have created a variety of aggregation operators that have been shown up to solve DM issues like Heronian aggregation operators [13], arithmetic aggregation operators [14], Hamacher aggregation operators [15], Dombi aggregation operators [16], [17] etc.

It's verified on the basis of evolved literature that most researchers prioritize fuzzy sets, interval-valued fuzzy sets, IFS, IVIFS, and their usage. Even so, Jun et al. [18] presented cubic sets (CSs) that merge interval-valued fuzzy data and fuzzy data, along with logic operations specified for them. Cubic hesitant fuzzy sets and their aggregation operators were introduced by Mahmood et al. [19] for applying them in decision-making. Khan et al. [20] initiated cubic aggregation operators. These theories not only address membership intervals primarily but also overlook the non-membership aspect, which is of equal significance in decision-making. It can actually get challenging to specify a membership function's value more precisely in a fuzzy set. Hence, describing vagueness and uncertainty in this situation may get easier using interval and exact values rather instead of focusing on the unique ones. As an outcome, when using an interval value and an exact number as a whole, one may find it simpler to show both confidence and unpredictability especially when proposing complex decisions and making tentative perceptions. Kaur and Garg [21] presented the notion of the cubic intuitionistic fuzzy set (CIFS) in order to handle it. When facing problems concerning interval-valued fuzzy data, we can label them using CIFS. We can effectively deal with such challenges by transforming data into CIFN format and setting values outside the interval to zero in both membership and non-membership degrees. Like, the transformation of an IVF number like ([0.3, 0.5], [0.1, 0.4]) into a CIFN format such as ([0.2, 0.6], 0), ([0.1, 0.3], 0) sorts out the adaptability of CIFS operators in handling incalculable problems. This lays stress on the superior capability of CIFS operators in mentioning a wide range of complex issues. It highlights the concept that blends two coinciding components. The first component consumes an interval-valued intuitionistic fuzzy value to demonstrate membership levels and the latter uses an intuitionistic fuzzy value to represent membership degrees. Simply, CIFS is composed of an IVIFN and an IFN. As CIFS can transfer both IVIFN and IFN at the same time by summarizing plenty of data, it can be seen that CIFS is highly advantageous. It holds a lot of information in contrast to the typical intuitionistic set (IVIFS/IFS) as a substitute because it gathers data from both sets. CIFS proves its practicality and ideality as a result when it approaches the alternatives in decision-making algorithms. This is all due to the chance that the traditional decision-making process may only use information from IVIFSs or IFSs, thereby excluding evaluative components from either IVIFS or IFS that could have an influence on desired outcomes.

Two main areas are focused on by the research on aggregation operators. The first one is the operational norms. Algebraic operational principles being a special case of Archimedean t-norm and t-conorm (ATT) [22], most aggregation operators using CIFS at present also use them [23]. The requirements of ATT are satisfied through the t-norms and t-conorms of SS [24]. However, SS operations are more adaptable than other existing approaches, because SS operations have a manageable parameter. Due to the adaptation ability to specific needs, users can be both optimistic and pessimistic in their conclusions assisting them in the efficient adjustment of risk by decision-makers. SS t-norms and t-conorms have been found appealing to many researchers due to their adaptability [25], [26], [27]. On the basis of these procedures, a number of fuzzy settings-based aggregating operators have been introduced consequently. We broaden the CIF weighted averaging and geometric operators using SS t-norms and t-conorms, inspired by the ideology of CFSs. The CIFSSPWA and CIFSSPWG operators being a family of influential aggregation operators are demonstrated by this research. Combining the power aggregation operator with SS operations, the data aggregation mechanism in the CIFS framework can be successfully increased by such operators.

### 1.1 Motivation

• Here we use a cubic intuitionistic fuzzy framework because it expresses the scenario in a more generalized way than an intuitionistic fuzzy framework. For example, we tackle a diabetes patient regarding his/her blood glucose level. During the monitoring period, their estimated blood glucose level is 120 mg/dL to 140 mg/dL in the normal state and 180 mg/dL to 200 mg/dL in the high state. At that time, his/her blood glucose level, which agrees with the normal state of 130 mg/dL and disagrees with the high state of 160 mg/dL, for this, the P-order cubic intuitionistic fuzzy set is (〈[120,140],130〉,〈[180,200],160〉). If his/her blood glucose level that disagrees with the normal state is 150 mg/dL and agrees with the high state is 190 mg/dL, then the R-order cubic intuitionistic fuzzy set is (〈[120,140],150〉,〈[180,200],190〉). Therefore, CIFS can successfully and independently convey the complexity within the decision-making process.
• The aggregation operators like CIFSSPWA and CIFSSPWG operators based on the SS operations are preferred because of the parameter β<0 and analyze how we can get more accuracy by changing the parameter *β*. These proposed operators provide more adaptability in real-world scenarios rather than IFSS power operators.

The leftover sections of this description are classified in the following pattern:

• The basic concepts of CIFSs are elaborated in Section 2. These contain an effective explanation of the idea, an outline of its different characters, and the correctness and scoring functions concerning CIFSs.
• In Section 3, the different aggregation operators based on SS operations along with their properties are demonstrated.
• Section 4 presents a sketch for employing CIF SS operators to sort out MCDM challenges.
• Section 5 gives an instance of how diabetes care has been revolutionized in the medical sector depicting the value of suggested accessing patterns.
• In Section 6, the constant *β* and characteristic weights are used to approach the sensitivity of the introduced operators.
• A comparative analysis between our recommended operators and the accessible possibilities is provided in Section 7.
• In Section 8, the article winds up and suggests some feasible directions for the future.

## 2 Fundamental ideas

Here, we mention some fundamental ideas concerning CIFSs.

Definition 1 [28] An *IFS* F on a given set *Ϝ* is described as

(1) $$F=\{<s,\tau_{F}(s),\nu_{F}(s)>:s\in Ϝ\},$$

where $0\leq \tau_{F}(s)\leq 1$, $0\leq \theta_{F}(s)\leq 1$ indicate the membership and non-membership degrees, correspondingly and $0\leq \tau_{F}(s)+\theta_{F}(s)\leq 1$.

Definition 2 [28] An IVIFS F on a given set *Ϝ* is defined as

(2) $$F=\{<s,[\tau_{F}^{lb}(s),\tau_{F}^{ub}(s)],[\theta_{F}^{lb}(s),\theta_{F}^{ub}(s)]>:s\in Ϝ\},$$

where $0\leq \tau_{F}^{lb}(s)\leq \tau_{F}^{ub}(s)\leq 1$, $0\leq \theta_{F}^{lb}(s)\leq \theta_{F}^{ub}(s)\leq 1$ and $0\leq \tau_{F}^{ub}(s)+\theta_{F}^{ub}(s)\leq 1$.

Definition 3 [29] An CIFS F on a given set *Ϝ* is defined as

(3) $$F=\{<s,\tau_{F}(s),\gamma_{F}(s)>:s\in Ϝ\},$$

where $\tau_{F}=\{<s,[\tau_{F}^{lb}(s),\tau_{F}^{ub}(s)],[\theta_{F}^{lb}(s),\theta_{F}^{ub}(s)]>:s\in Ϝ\}$ represent the IVIFS on *Ϝ* and $\gamma_{F}=\{<s,\varepsilon_{F}(s),\zeta_{F}(s)>:s\in Ϝ\}$ represent the *IFS* on *Ϝ*. And $<[\tau_{F}^{lb}(s),\tau_{F}^{ub}(s)],\varepsilon_{F}(s)>$ represent the membership grade and $<[\theta_{F}^{lb}(s),\theta_{F}^{ub}(s)],\zeta_{F}(s)>$ represent the non-membership grade of F such that ε+ζ≤1 and $\tau^{ub}+\theta^{ub}\leq 1$. Furthermore, CIFS hesitation margin of s∈Ϝ is $\pi (F)=\{[1-(\tau^{lb}+\theta^{lb}),1-(\tau^{ub}+\theta^{ub})],1-(\varepsilon +\zeta )\}$. The pair $\left([\tau^{lb},\tau^{ub}],\varepsilon ,[\theta^{lb},\theta^{ub}],\zeta \right)$ is called CIFN and denoted by *σ*.

Definition 4 [29] Suppose $\sigma =(<[\tau^{lb},\tau^{ub}],\varepsilon >,<[\theta^{lb},\theta^{ub}],\zeta >)$ be a CIFN. The Score function of F is indicated by S(F) and then defined as

(4) $$S(\varrho )=\{(\tau^{lb}+\tau^{ub}+\varepsilon -\theta^{lb}+\theta^{ub}-\zeta )/3\}$$

such that S(σ)∈[−1,1].

Definition 5 [29] Suppose $\sigma =(<[\tau^{lb},\tau^{ub}],\varepsilon >,<[\theta^{lb},\theta^{ub}],\zeta >)$ be a CIFN. The Accuracy function of F is indicated by ℜ(F) and thus defined as

(5) $$ℜ(\varrho )=\{(\tau^{lb}+\tau^{ub}+\varepsilon +\theta^{lb}+\theta^{ub}+\zeta )/3\}$$

such that ℜ(ϱ)∈[0,1].

Definition 6 [29] Suppose $\varrho_{1}=(<[\tau_{1}^{lb},\tau_{1}^{ub}],\varepsilon_{1}>,<[\theta_{1}^{lb},\theta_{1}^{ub}],\zeta_{1}>)$ and $\varrho_{2}=(<[\tau_{2}^{lb},\tau_{2}^{ub}],\varepsilon_{2}>,<[\theta_{2}^{lb},\theta_{2}^{ub}],\zeta_{2}>)$ are two CIFNs, then

$S(\varrho_{1})<S(\varrho_{2})\Rightarrow \varrho_{1}<\varrho_{2}$.

Definition 7 [30] For any CIFNs $\varrho =(<[\tau^{lb},\tau^{ub}],\varepsilon >,<[\theta^{lb},\theta^{ub}],\zeta >)$ and $\varrho_{i}=(<[\tau_{i}^{lb},\tau_{i}^{ub}],\varepsilon_{i}>,<[\theta_{i}^{lb},\theta_{i}^{ub}],\zeta_{i}>)$, i∈F, the following operations hold:

**(i)** $\varrho^{c}=(<[\theta^{lb},\theta^{ub}],\zeta >,<[\tau^{lb},\tau^{ub}],\varepsilon >)$;
**(ii)** $\varrho_{1}=\varrho_{2}\Leftrightarrow [\tau_{1}^{lb},\tau_{1}^{ub}]=[\tau_{2}^{lb},\tau_{2}^{ub}],[\theta_{1}^{lb},\theta_{1}^{ub}]=[\theta_{2}^{lb},\theta_{2}^{ub}],\varepsilon_{1}=\varepsilon_{2}$ and $\zeta_{1}=\zeta_{2}$;
**(iii)** $\varrho_{1}\subseteq \varrho_{2}\Leftrightarrow [\tau_{1}^{lb},\tau_{1}^{ub}]\subseteq [\tau_{2}^{lb},\tau_{2}^{ub}],[\theta_{1}^{lb},\theta_{1}^{ub}]⊇[\theta_{2}^{lb},\theta_{2}^{ub}],\varepsilon_{1}\geq \varepsilon_{2}$ and $\zeta_{1}\leq \zeta_{2}$;
**(iv)** $⋃\varrho_{i}=(\langle [\sup_{i\in F}\tau_{i}^{lb},\sup_{i\in F}\tau_{i}^{ub}],\inf_{i\in F}\varepsilon_{i}\rangle ,\langle [\inf_{i\in F}\theta_{i}^{lb},\inf_{i\in F}\theta_{i}^{ub}],\sup_{i\in F}\zeta_{i}\rangle )$;
**(v)** $⋂\varrho_{i}=(\langle [\inf_{i\in F}\tau_{i}^{lb},\inf_{i\in F}\tau_{i}^{ub}],\sup_{i\in F}\varepsilon_{i}\rangle ,\langle [\sup_{i\in F}\theta_{i}^{lb},\sup_{i\in F}\theta_{i}^{ub}],\inf_{i\in F}\zeta_{i}\rangle )$;

Garg and Kaur [30] proposed cubic intuitionistic fuzzy distances to measure the variation between two CIFNs.

Definition 8 Suppose $\varrho_{1}=(<[\tau_{1}^{lb},\tau_{1}^{ub}],\varepsilon_{1}>,<[\theta_{1}^{lb},\theta_{1}^{ub}],\zeta_{1}>)$ and $\varrho_{2}=(<[\tau_{2}^{lb},\tau_{2}^{ub}],\varepsilon_{2}>,<[\theta_{2}^{lb},\theta_{2}^{ub}],\zeta_{2}>)$ any two CIFNs. The normalized Hamming distance between $\varrho_{1}$ and $\varrho_{2}$, denoted as $d(\varrho_{1},\varrho_{2})$, are then defined as

(6) $$d(\varrho_{1},\varrho_{2})=\frac{\left(|\tau_{1}^{lb}-\tau_{2}^{lb}|+|\tau_{1}^{ub}-\tau_{2}^{ub}|+|\varepsilon_{1}-\varepsilon_{2}|)+(|\theta_{1}^{lb}-\theta_{2}^{lb}|+|\theta_{1}^{ub}-\theta_{2}^{ub}|+|\zeta_{1}+\zeta_{2}|\right)}{6}$$

and it is used to calculate the supports (similar index) through the formula $\kappa (\varrho_{1},\varrho_{2})=1-d(\varrho_{1},\varrho_{2})$ [31].

Yager [32] proposed the power average operator that enabled input values to support one another in the aggregation step where we used operations based on SS t-norms and t-conorms.

Definition 9 Assume that $\varrho_{t},(t=1,2,...,n)$ is the collection of CIFNs. Then, the Power Average operator (PA) is given as

(7) $$PA(\varrho_{1},\varrho_{2},...,\varrho_{n})=\frac{\sum_{t=1}^{n}(1+ℸ(\varrho_{t}))\varrho_{t}}{\sum_{t=1}^{n}(1+ℸ(\varrho_{t})},$$

where $ℸ(\varrho_{t})=\sum_{t=1,t\neq r}^{n}\kappa (\varrho_{t},\varrho r)$ and $\kappa (\varrho_{t},\varrho r)$ represented the support for $\varrho_{t}$ from $\varrho_{r}$ satisfying:

• $\kappa (\varrho_{t},\varrho_{r})\in [0,1]$;
• $\kappa (\varrho_{t},\varrho_{r})=\kappa (\varrho_{r},\varrho_{t})$;
• $\kappa (\varrho_{t},\varrho_{r})\geq \kappa (\varrho_{j},\varrho_{s})$, if $\left|\varrho_{t}-\varrho_{r}|<|\varrho_{j}-\varrho_{s}\right|$, where the distance between two CIFNs is represented by |.|.

Xu and Yager [33] proposed the PG operator based on the power average operator and geometric mean.

Definition 10 Assume that $\varrho_{t},(t=1,2,...,n)$ is the collection of CIFNs. Then, the (PG) Power Geometric operator is provided as

(8) $$PG(\varrho_{1},\varrho_{2},...,\varrho_{n})=\prod_{t=1}^{n}\varrho_{t}^{\frac{1+ℸ(\varrho_{t})}{\sum_{t=1}^{n}(1+ℸ(\varrho_{t})}},$$

where the idea gives the same meaning as discussed before.

Definition 11 [34] The t-norms and t-conorms for SS can be obtained by

• $T(m,n)={\left\{m^{\beta }+n^{\beta }-1\right\}}^{1/\beta }$,
• $S(m,n)=1-{\left\{{\left(1-m\right)}^{\beta }+{\left(1-n\right)}^{\beta }-1\right\}}^{1/\beta }$, correspondingly, where m,n∈[0,1] and β<0.

Besides, if β=0, then algebraic t-norms and t-conorms substitute SS t-norms and t-conorms. When β>0, then *T* and *S* do not satisfy the closure as well as the monotonicity property.

## 3 Deployment of cubic intuitionistic fuzzy power aggregation operators based on SS operations

Here, we introduce CIFSSPWA and CIFSSPWG operators based on SS t-norms and t-conorms, respectively.

Definition 12 Suppose $\varrho_{1}=(<[\tau_{1}^{lb},\tau_{1}^{ub}],\varepsilon_{1}>,<[\theta_{1}^{lb},\theta_{1}^{ub}],\zeta_{1}>)$ and $\varrho_{2}=(<[\tau_{2}^{lb},\tau_{2}^{ub}],\varepsilon_{2}>,<[\theta_{2}^{lb},\theta_{2}^{ub}],\zeta_{2}>)$ be two CIFNs with constraints β<0 and c>0. Then, SS operating rules according to t-norms and t-conorms for SS are stated below:

$$\varrho_{1}\underset{SS}{⨁}\varrho_{2}=(\langle \left[1-{\left\{{\left(1-\tau_{1}^{lb}\right)}^{\beta }+{\left(1-\tau_{2}^{lb}\right)}^{\beta }-1\right\}}^{1/\beta },1-{\left\{{\left(1-\tau_{1}^{ub}\right)}^{\beta }+{\left(1-\tau_{2}^{ub}\right)}^{\beta }-1\right\}}^{1/\beta }\right]\;,1-{\left\{{\left(1-\varepsilon_{1}\right)}^{\beta }+{\left(1-\varepsilon_{2}\right)}^{\beta }-1\right\}}^{1/\beta }\rangle \;,\langle \left[{\left\{{\left(\theta_{1}^{lb}\right)}^{\beta }+{\left(\theta_{2}^{lb}\right)}^{\beta }-1\right\}}^{1/\beta },{\left\{{\left(\theta_{1}^{ub}\right)}^{\beta }+{\left(\theta_{2}^{ub}\right)}^{\beta }-1\right\}}^{1/\beta }\right],{\left\{{\left(\zeta_{1}\right)}^{\beta }+{\left(\zeta_{2}\right)}^{\beta }-1\right\}}^{1/\beta }\rangle );$$

$$c\varrho_{1}=(\langle \left[1-{\left\{c{\left(1-\tau_{1}^{lb}\right)}^{\beta }\right\}}^{1/\beta },1-{\left\{c{\left(1-\tau_{1}^{ub}\right)}^{\beta }\right\}}^{1/\beta }\right],1-{\left\{c{\left(1-\varepsilon_{1}\right)}^{\beta }\right\}}^{1/\beta }\rangle \;,\langle \left[{\left\{c{\left(\theta_{1}^{lb}\right)}^{\beta }-(c-1)\right\}}^{1/\beta },{\left\{c{\left(\theta_{1}^{ub}\right)}^{\beta }-(c-1)\right\}}^{1/\beta }\right],{\left\{c{\left(\zeta_{1}\right)}^{\beta }-(c-1)\right\}}^{1/\beta }\rangle );$$

$$\varrho_{1}\underset{SS}{⨂}\varrho_{2}=(\langle \left[{\left\{{\left(\tau_{1}^{lb}\right)}^{\beta }+{\left(\tau_{2}^{lb}\right)}^{\beta }-1\right\}}^{1/\beta },{\left\{{\left(\tau_{1}^{ub}\right)}^{\beta }+{\left(\tau_{2}^{ub}\right)}^{\beta }-1\right\}}^{1/\beta }\right],{\left\{{\left(\varepsilon_{1}\right)}^{\beta }+{\left(\varepsilon_{2}\right)}^{\beta }-1\right\}}^{1/\beta }\rangle \;,\langle \left[1-{\left\{{\left(1-\theta_{1}^{lb}\right)}^{\beta }+{\left(1-\theta_{2}^{lb}\right)}^{\beta }-1\right\}}^{1/\beta },1-{\left\{{\left(1-\theta_{1}^{ub}\right)}^{\beta }+{\left(1-\theta_{2}^{ub}\right)}^{\beta }-1\right\}}^{1/\beta }\right]\;,1-{\left\{{\left(1-\zeta_{1}\right)}^{\beta }+{\left(1-\zeta_{2}\right)}^{\beta }-1\right\}}^{1/\beta }\rangle );$$

$$\varrho_{1}^{c}=(\langle \left[{\left\{c{\left(\tau_{1}^{lb}\right)}^{\beta }-(c-1)\right\}}^{1/\beta },{\left\{c{\left(\tau_{1}^{ub}\right)}^{\beta }-(c-1)\right\}}^{1/\beta }\right],{\left\{c{\left(\varepsilon_{1}\right)}^{\beta }-(c-1)\right\}}^{1/\beta }\rangle \;,\langle \left[1-{\left\{c{\left(1-\theta_{1}^{lb}\right)}^{\beta }\right\}}^{1/\beta },1-{\left\{c{\left(1-\theta_{1}^{ub}\right)}^{\beta }\right\}}^{1/\beta }\right],1-{\left\{c{\left(1-\zeta_{1}\right)}^{\beta }\right\}}^{1/\beta }\rangle );$$

Theorem 1 *Suppose*$\varrho_{1}=(<[\tau_{1}^{lb},\tau_{1}^{ub}],\varepsilon_{1}>,<[\theta_{1}^{lb},\theta_{1}^{ub}],\zeta_{1}>)$*and*$\varrho_{2}=(<[\tau_{2}^{lb},\tau_{2}^{ub}],\varepsilon_{2}>,<[\theta_{2}^{lb},\theta_{2}^{ub}],\zeta_{2}>)$*be two*CIFNs*with constraints*β<0*and*$n,n_{1},n_{2}\geq 0$*. Then, according to*Definition 12*, some following properties hold:*

**(i)** $\varrho_{1}{⨁}_{SS}\varrho_{2}=\varrho_{2}{⨁}_{SS}\varrho_{1}$*;*
**(ii)** $\varrho_{1}{⨂}_{SS}\varrho_{2}=\varrho_{2}{⨂}_{SS}\varrho_{1}$*;*
**(iii)** $n(\varrho_{1}{⨁}_{SS}\varrho_{2})=n\varrho_{1}{⨁}_{SS}n\varrho_{2}$*;*
**(iv)** $n_{1}\varrho_{1}{⨁}_{SS}n_{2}\varrho_{1}=(n_{1}+n_{2})\varrho_{1}$*;*
**(v)** $\varrho_{1}^{n_{1}}⨂\varrho_{1}^{n_{2}}=\varrho_{1}^{n_{1}+n_{2}}$*;*
**(vi)** $\varrho_{1}^{n_{1}}⨂\varrho_{2}^{n_{1}}={\left(\varrho_{1}⨂\varrho_{2}\right)}^{n_{1}}$*;*

Proof Theorem [1] is readily proven. □

### 3.1 Cubic intuitionistic fuzzy SS power weight average aggregation operator

Definition 13 Suppose that $\overset{˜}{\varpi }={\left(\varpi_{1},\varpi_{2},...,\varpi_{n}\right)}^{T}$ is the weight vector wherein $\varpi_{t}\in [0,1]$ for t∈{1,2,...,n} such that $\sum_{t=1}^{n}\varpi_{t}=1$. Suppose, there are $\varrho_{t},(t=1,2,...,n)$ CIFNs in this array. Then, a mapping $Z^{n}\times Z^{n}$ → Z×Z is shown as the CIFSSPWA operator, in order that

(9) $$CIFSSPWA(\varrho_{1},\varrho_{2},...,\varrho_{n})=\frac{{\underset{SS}{⨁}}_{t=1}^{n}(\varpi_{t}\{1+ℸ(\varrho_{t})\}\varrho_{t})}{\sum_{t=1}^{n}\varpi_{t}\{1+ℸ(\varrho_{t})\}},$$

where $ℸ(\varrho_{t})=\sum_{t=1,t\neq r}^{n}\kappa (\varrho_{t},\varrho r)$ and $\kappa (\varrho_{t},\varrho r)$ indicated the support for $\varrho_{t}$ from $\varrho_{r}$. By using the definition [13], the next theorem shows that the aggregate value of CIFNs is likewise a CIFN.

Theorem 2 *Let* $\overset{˜}{\varpi }={\left(\varpi_{1},\varpi_{2},...,\varpi_{n}\right)}^{T}$ *be the weight vector wherein* $\varpi_{t}\in [0,1]$ *for* t∈{1,2,...,n} *such that* $\sum_{t=1}^{n}\varpi_{t}=1$ *. Suppose, there are* $\varrho_{t},(t=1,2,...,n)$ CIFNs *in this array. Then, using* CIFSSPWA *operator, the aggregate yield remains a* CIFN *, satisfying*

(10) $$CIFSSPWA(\varrho_{1},\varrho_{2},...,\varrho_{n})=\frac{{\underset{SS}{⨁}}_{t=1}^{n}(\varpi_{t}\{1+ℸ(\varrho_{t})\}\varrho_{t})}{\sum_{t=1}^{n}\varpi_{t}\{1+ℸ(\varrho_{t})\}}=(\langle \left[1-{\left\{\sum_{t=1}^{n}\chi_{t}{\left(1-\tau_{t}^{lb}\right)}^{\beta }\right\}}^{1/\beta },1-{\left\{\sum_{t=1}^{n}\chi_{t}{\left(1-\tau_{t}^{ub}\right)}^{\beta }\right\}}^{1/\beta }\right],1-{\left\{\sum_{t=1}^{n}\chi_{t}{\left(1-\varepsilon_{t}\right)}^{\beta }\right\}}^{1/\beta }\rangle \;,\langle \left[{\left\{\sum_{t=1}^{n}\chi_{t}{\left(\theta_{t}^{lb}\right)}^{\beta }\right\}}^{1/\beta },{\left\{\sum_{t=1}^{n}\chi_{t}{\left(\theta_{t}^{ub}\right)}^{\beta }\right\}}^{1/\beta }\right],{\left\{\sum_{t=1}^{n}\chi_{t}{\left(\zeta_{t}\right)}^{\beta }\right\}}^{1/\beta }\rangle )$$

*where* $\chi_{t}=\frac{\varpi_{t}\{1+ℸ(\varrho_{t})\}}{\sum_{t=1}^{n}\varpi_{t}\{1+ℸ(\varrho_{t})\}},(t=1,2,...,n)$ *and* $ℸ(\varrho_{t})=\sum_{t=1,t\neq r}^{n}\kappa (\varrho_{t},\varrho r)$ *.*

Proof First, we are to show the following result.

For any $\chi ={\left(\chi_{1},\chi_{2},...,\chi_{n}\right)}^{T}$,

(11) $$CIFSSPWA(\varrho_{1},\varrho_{2},\ldots ,\varrho_{n})=(\langle [1-{\left\{\sum_{t=1}^{n}\chi_{t}{\left(1-\tau_{t}^{lb}\right)}^{\beta }-\sum_{t=1}^{n}\chi_{t}+1\right\}}^{1/\beta },1-{\left\{\sum_{t=1}^{n}\chi_{t}{\left(1-\tau_{t}^{ub}\right)}^{\beta }-\sum_{t=1}^{n}\chi_{t}+1\right\}}^{1/\beta }],1-{\left\{\sum_{t=1}^{n}\chi_{t}{\left(1-\varepsilon_{t}\right)}^{\beta }-\sum_{t=1}^{n}\chi_{t}+1\right\}}^{1/\beta }\rangle \;,\langle \left[{\left\{\sum_{t=1}^{n}\chi_{t}{\left(\theta_{t}^{lb}\right)}^{\beta }-\sum_{t=1}^{n}\chi_{t}+1\right\}}^{1/\beta },{\left\{\sum_{t=1}^{n}\chi_{t}{\left(\theta_{t}^{ub}\right)}^{\beta }-\sum_{t=1}^{n}\chi_{t}+1\right\}}^{1/\beta }\right],{\left\{\sum_{t=1}^{n}\chi_{t}{\left(\zeta_{t}\right)}^{\beta }-\sum_{t=1}^{n}\chi_{t}+1\right\}}^{1/\beta }\rangle )$$

The method of induction is used to demonstrate the given equation.

Take t=2, then

$$CIFSSPWA(\varrho_{1},\varrho_{2})=\frac{{\underset{SS}{⨁}}_{t=1}^{2}(\chi_{t}\{1+ℸ(\varrho_{t})\}\varrho_{t})}{\sum_{t=1}^{2}\chi_{t}\{1+ℸ(\varrho_{t})\}}=(\langle \left[1-{\left(\sum_{t=1}^{2}\chi_{t}{\left\{1-\tau_{t}^{lb}\right\}}^{\beta }-\sum_{t=1}^{2}\chi_{t}+1\right)}^{1/\beta },1-{\left(\sum_{t=1}^{2}\chi_{t}{\left\{1-\tau_{t}^{ub}\right\}}^{\beta }-\sum_{t=1}^{2}\chi_{t}+1\right)}^{1/\beta }\right],\;1-{\left(\sum_{t=1}^{2}\chi_{t}{\left\{1-\varepsilon_{t}\right\}}^{\beta }-\sum_{t=1}^{2}\chi_{t}+1\right)}^{1/\beta }\rangle ,\;\langle \left[{\left(\sum_{t=1}^{2}\chi_{t}{\left(\theta_{t}^{lb}\right)}^{\beta }-\sum_{t=1}^{2}\chi_{t}+1\right)}^{1/\beta },{\left(\sum_{t=1}^{2}\chi_{t}{\left(\theta_{t}^{ub}\right)}^{\beta }-\sum_{t=1}^{2}\chi_{t}+1\right)}^{1/\beta }\right],\;{\left(\sum_{t=1}^{2}\chi_{t}{\left(\zeta_{t}\right)}^{\beta }-\sum_{t=1}^{2}\chi_{t}+1\right)}^{1/\beta }\rangle )$$

Thus, for t=2, it is a true result.

Assume that statement is true for t=m, let's proceed.

$$CIFSSPWA(\varrho_{1},\varrho_{2},...,\varrho_{m})=(\langle [1-{\left\{\sum_{t=1}^{m}\chi_{t}{\left(1-\tau_{t}^{lb}\right)}^{\beta }-\sum_{t=1}^{m}\chi_{t}+1\right\}}^{1/\beta },\;1-{\left\{\sum_{t=1}^{m}\chi_{t}1-{\left(\tau_{t}^{ub}\right)}^{\beta }-\sum_{t=1}^{m}\chi_{t}+1\right\}}^{1/\beta }],1-{\left\{\sum_{t=1}^{m}\chi_{t}{\left(1-\varepsilon_{t}\right)}^{\beta }-\sum_{t=1}^{m}\chi_{t}+1\right\}}^{1/\beta }\rangle ,\;\langle \left[{\left\{\sum_{t=1}^{m}\chi_{t}{\left(\theta_{t}^{lb}\right)}^{\beta }-\sum_{t=1}^{m}\chi_{t}+1\right\}}^{1/\beta },{\left\{\sum_{t=1}^{m}\chi_{t}{\left(\theta_{t}^{ub}\right)}^{\beta }-\sum_{t=1}^{m}\chi_{t}+1\right\}}^{1/\beta }\right],{\left\{\sum_{t=1}^{m}\chi_{t}{\left(\zeta_{t}\right)}^{\beta }-\sum_{t=1}^{m}\chi_{t}+1\right\}}^{1/\beta }\rangle )$$

Choosing t=m+1 now, proceed by [12],

$$\chi_{m+1}\varrho_{m+1}=(\langle [1-{\left\{\chi_{m+1}{\left(1-\tau_{m+1}^{lb}\right)}^{\beta }-(\chi_{m+1}-1)\right\}}^{1/\beta }\;\;,1-{\left\{\chi_{m+1}{\left(1-\tau_{m+1}^{ub}\right)}^{\beta }-(\chi_{m+1}+1)\right\}}^{1/\beta }],1-{\left\{\chi_{m+1}{\left(1-\varepsilon_{m+1}\right)}^{\beta }-(\chi_{m+1}+1)\right\}}^{1/\beta }\rangle \;,\langle \left[{\left\{\chi_{m+1}{\left(\theta_{m+1}^{lb}\right)}^{\beta }-(\chi_{m+1}-1)\right\}}^{1/\beta },{\left\{\chi_{m+1}{\left(\theta_{m+1}^{ub}\right)}^{\beta }-(\chi_{m+1}-1)\right\}}^{1/\beta }\right],{\left\{\chi_{m+1}{\left(\zeta_{m+1}\right)}^{\beta }-(\chi_{m+1}-1)\right\}}^{1/\beta }\rangle )$$

$$CIFSSPWA(\varrho_{1},\varrho_{2},...,\varrho_{m+1})=CIFSSPWA(\varrho_{1},\varrho_{2},...,\varrho_{m})⨁\chi_{m+1}\varrho_{m+1}=(\langle [1-{\left\{{\left(1-\{1-{\left(\sum_{t=1}^{m}\chi_{t}{\left(1-\tau_{t}^{lb}\right)}^{\beta }-\sum_{t=1}^{m}\chi_{t}+1\right)}^{1/\beta }\}\right)}^{\beta }+{\left(1-\{1-{\left(\chi_{m+1}{\left(1-\tau_{m+1}^{lb}\right)}^{\beta }-(\chi_{m+1}-1)\right)}^{1/\beta })\right\}}^{\beta }-1\right\}}^{1/\beta }\;\;,1-\{{\left(1-\{1-{\left(\sum_{t=1}^{m}\chi_{t}{\left(1-\theta_{t}^{lb}\right)}^{\beta }-\sum_{t=1}^{m}\chi_{t}+1\right)}^{1/\beta }\}\right)}^{\beta }+{\left(1-{\left\{1-(\chi_{m+1}{\left(1-\tau_{m+1}^{ub}\right)}^{\beta }-{\left(\chi_{m+1}-1\right)}^{1/\beta })\right\}}^{\beta }-1\right\}}^{1/\beta }]\;,1-{\left\{{\left(1-\{1-{\left(\sum_{t=1}^{m}\chi_{t}{\left(1-\varepsilon_{t}\right)}^{\beta }-\sum_{t=1}^{m}\chi_{t}+1\right)}^{1/\beta }\}\right)}^{\beta }+{\left(1-\{1-{\left(\chi_{m+1}{\left(1-\varepsilon_{m+1}\right)}^{\beta }-(\chi_{m+1}-1)\right)}^{1/\beta })\right\}}^{\beta }-1\right\}}^{1/\beta }\rangle \;,\langle [{\left\{(\sum_{t=1}^{m}\chi_{t}{\left(\theta_{t}^{lb}\right)}^{\beta }-\sum_{t=1}^{m}\chi_{t}+1)+(\chi_{m+1}{\left(\theta_{m+1}^{lb}\right)}^{\beta }-(\chi_{m+1}-1))-1\right\}}^{1/\beta }\;\;,{\left\{(\sum_{t=1}^{m}\chi_{t}{\left(\theta_{t}^{ub}\right)}^{\beta }-\sum_{t=1}^{m}\chi_{t}+1)+(\chi_{m+1}{\left(\theta_{m+1}^{ub}\right)}^{\beta }-(\chi_{m+1}-1))-1\right\}}^{1/\beta }]\;,{\left\{(\sum_{t=1}^{m}\chi_{t}{\left(\zeta_{t}\right)}^{\beta }-\sum_{t=1}^{m}\chi_{t}+1)+(\chi_{m+1}{\left(\zeta_{m+1}\right)}^{\beta }-(\chi_{m+1}-1))-1\right\}}^{1/\beta }\rangle )=(\langle [1-{\left\{\sum_{t=1}^{m+1}\chi_{t}{\left(1-\tau_{t}^{lb}\right)}^{\beta }-\sum_{t=1}^{m+1}\chi_{t}+1\right\}}^{1/\beta }\;\;,1-\{\sum_{t=1}^{m+1}\chi_{t}{\left(1-\tau_{t}^{ub}{}^{\beta }-\sum_{t=1}^{m+1}\chi_{t}+1\right\}}^{1/\beta }],1-{\left\{\sum_{t=1}^{m+1}\chi_{t}{\left(1-\varepsilon_{t}\right)}^{\beta }-\sum_{t=1}^{m+1}\chi_{t}+1\right\}}^{1/\beta }\rangle \;,\langle \left[{\left\{\sum_{t=1}^{m+1}\chi_{t}{\left(\theta_{t}^{lb}\right)}^{\beta }-\sum_{t=1}^{m+1}\chi_{t}+1\right\}}^{1/\beta },{\left\{\sum_{t=1}^{m+1}\chi_{t}{\left(\theta_{t}^{ub}\right)}^{\beta }-\sum_{t=1}^{m+1}\chi_{t}+1\right\}}^{1/\beta }\right]\;,{\left\{\sum_{t=1}^{m+1}\chi_{t}{\left(\zeta_{t}\right)}^{\beta }-\sum_{t=1}^{m+1}\chi_{t}+1\right\}}^{1/\beta }\rangle )$$

Thus, the outcome is true for t=m+1.

Since the claim holds for every value of *χ*, it also holds when the subsequent conditions are met: with $\sum_{t=1}^{n}\chi_{t}=1$, $\chi_{t}\geq 0$.

Hence, the theorem has been proven. □

Theorem 3 *Let* F *be a CFS on a given set Ϝ. The following properties fulfill:*

**(i)** *[Idempotency] For every* t=1,2,...,n *, assume that* $\overset{˜}{\varpi }={\left(\varpi_{1},\varpi_{2},...,\varpi_{n}\right)}^{T}$ *is weight vector wherein* $\varpi_{t}\in [0,1]$ *, and if all* $\varrho_{t}=([\tau_{t}^{lb},\tau_{t}^{ub}],\varepsilon_{t}>,[\theta_{t}^{lb},\theta_{t}^{ub}],\zeta_{t}>)$ *are equal, then*
  $$CIFSSPWA(\varrho_{1},\varrho_{2},...,\varrho_{n})=\varrho .$$
**(ii)** *[Monotonicity] Assume that* $\varrho_{t}=(<[\tau_{t}^{lb},\tau_{t}^{ub}],\varepsilon_{t}>,<[\theta_{t}^{lb},\theta_{t}^{ub}],\zeta_{t}>)$ *and* $\varrho_{t}^{\prime }=(<[{\left(\tau^{lb}\right)}_{t}^{\prime },{\left(\tau^{ub}\right)}_{t}^{\prime }],\varepsilon_{t}^{\prime }>,<[{\left(\theta^{lb}\right)}_{t}^{\prime },{\left(\theta^{ub}\right)}_{t}^{\prime }],\zeta_{t}^{\prime }>)$ *,* (t=1,2,...,n) *as two arrays of* CIFNs *. Also assume that* $\overset{˜}{\varpi }={\left(\varpi_{1},\varpi_{2},...,\varpi_{n}\right)}^{T}$ *is weight vector wherein* $\varpi_{t}\in [0,1]$ *such that* $\tau_{t}^{lb}\leq {\left(\tau^{lb}\right)}_{t}^{\prime }$ *,* $\tau_{t}^{ub}\leq {\left(\tau^{ub}\right)}_{t}^{\prime }$ *,* $\varepsilon_{t}\leq {\left(\varepsilon \right)}_{t}^{\prime }$ *;* $\theta_{t}^{lb}\geq {\left(\theta^{lb}\right)}_{t}^{\prime }$ *,* $\theta_{t}^{ub}\geq {\left(\theta^{ub}\right)}_{t}^{\prime }$ *,* $\zeta_{t}\geq {\left(\zeta \right)}_{t}^{\prime }$ *for all* t∈1,2,...,n *, then*
  $$CIFSSPWA(\varrho_{1},\varrho_{2},...,\varrho_{t})\leq CIFSSPWA(\varrho_{1}^{\prime },\varrho_{2}^{\prime },...,\varrho_{t}^{\prime }).$$
**(iii)** *[Boundedness] Consider* $\varrho_{t}=(<[\tau_{t}^{lb},\tau_{t}^{ub}],\varepsilon_{t}>,<[\theta_{t}^{lb},\theta_{t}^{ub}],\zeta_{t}>)$ *be a set of* CIFNs *. Also assume that* $\overset{˜}{\varpi }={\left(\varpi_{1},\varpi_{2},...,\varpi_{n}\right)}^{T}$ *is weight vector wherein* $\varpi_{t}\in [0,1]$ *, then*
  $$\varrho_{min}\leq CIFSSPWA(\varrho_{1},\varrho_{2},...,\varrho_{t})\leq \varrho_{max}$$
  *where* $\varrho_{min}=\min_{t}\{\varrho_{t}\}$ *and* $\varrho_{max}=\max_{t}\{\varrho_{t}\}$ *.*

Proof

**(i)** $CIFSSPWA(\varrho_{1},\varrho_{2},...,\varrho_{n})=(\langle \left[1-{\left\{\sum_{t=1}^{n}\chi_{t}{\left(1-\tau_{t}^{lb}\right)}^{\beta }\right\}}^{1/\beta },1-{\left\{\sum_{t=1}^{n}\chi_{t}{\left(1-\tau_{t}^{ub}\right)}^{\beta }\right\}}^{1/\beta }\right],$
$\;1-{\left\{\sum_{t=1}^{n}\chi_{t}{\left(1-\varepsilon_{t}\right)}^{\beta }\right\}}^{1/\beta }\rangle ,\langle \left[{\left\{\sum_{t=1}^{n}\chi_{t}{\left(\theta_{t}^{lb}\right)}^{\beta }\right\}}^{1/\beta },{\left\{\sum_{t=1}^{n}\chi_{t}{\left(\theta_{t}^{ub}\right)}^{\beta }\right\}}^{1/\beta }\right],{\left\{\sum_{t=1}^{n}\chi_{t}{\left(\zeta_{t}\right)}^{\beta }\right\}}^{1/\beta }\rangle )$
$=(\langle \left[1-{\left\{\sum_{t=1}^{n}\chi_{t}{\left(1-\tau^{lb}\right)}^{\beta }\right\}}^{1/\beta },1-{\left\{\sum_{t=1}^{n}\chi_{t}{\left(1-\tau^{ub}\right)}^{\beta }\right\}}^{1/\beta }\right],1-{\left\{\sum_{t=1}^{n}\chi_{t}{\left(1-\varepsilon \right)}^{\beta }\right\}}^{1/\beta }\rangle ,$
$\;\langle \left[{\left\{\sum_{t=1}^{n}\chi_{t}{\left(\theta^{lb}\right)}^{\beta }\right\}}^{1/\beta },{\left\{\sum_{t=1}^{n}\chi_{t}{\left(\theta^{ub}\right)}^{\beta }\right\}}^{1/\beta }\right],{\left\{\sum_{t=1}^{n}\chi_{t}{\left(\zeta \right)}^{\beta }\right\}}^{1/\beta }\rangle )$
  $=(<[\tau_{t}^{lb},\tau_{t}^{ub}],\varepsilon_{t}>,<[\theta_{t}^{lb},\theta_{t}^{ub}],\zeta_{t}>)=\varrho$.
**(ii)** Since $\tau_{t}^{lb}\leq {\left(\tau^{lb}\right)}_{t}^{\prime }$ and $\theta_{t}^{lb}\geq {\left(\theta^{lb}\right)}_{t}^{\prime }$,
  $\Rightarrow {\left(1-\tau_{t}^{lb}\right)}^{\beta }\leq {\left(1-{\left(\tau^{lb}\right)}_{t}^{\prime }\right)}^{\beta }$, (since β<0)
  ${{\Rightarrow \{\sum_{t=1}^{n}\chi_{t}^{\prime }{\left(1-{\left(\tau^{lb}\right)}_{t}^{\prime }\right)}^{\beta })\}}^{1/\beta }\leq \{\sum_{t=1}^{n}\chi_{t}{\left(1-\tau_{t}^{lb}\right)}^{\beta })\}}^{1/\beta }$,
${{\Rightarrow 1-\{\sum_{t=1}^{n}\chi_{t}{\left(1-\tau_{t}^{lb}\right)}^{\beta })\}}^{1/\beta }\leq 1-\{\sum_{t=1}^{n}\chi_{t}^{\prime }{\left(1-{\left(\tau^{lb}\right)}_{t}^{\prime }\right)}^{\beta })\}}^{1/\beta }$
  and ${\left\{\sum_{t=1}^{n}\chi_{t}^{\prime }({\left(\theta^{lb}\right)}_{t}^{\prime })\right\}}^{\beta }\leq {\left\{\sum_{t=1}^{n}\chi_{t}(\theta_{t}^{lb})\right\}}^{\beta }$ and ${\left\{\sum_{t=1}^{n}\chi_{t}^{\prime }({\left(\varepsilon \right)}_{t}^{\prime })\right\}}^{\beta }\leq {\left\{\sum_{t=1}^{n}\chi_{t}(\varepsilon_{t})\right\}}^{\beta }$.
  Similarly, we have
${{\Rightarrow 1-\{\sum_{t=1}^{n}\chi_{t}{\left(1-\tau_{t}^{ub}\right)}^{\beta })\}}^{1/\beta }\leq 1-\{\sum_{t=1}^{n}\chi_{t}^{\prime }{\left(1-{\left(\tau^{ub}\right)}_{t}^{\prime }\right)}^{\beta })\}}^{1/\beta }$
  and ${\left\{\sum_{t=1}^{n}\chi_{t}^{\prime }({\left(\theta^{ub}\right)}_{t}^{\prime })\right\}}^{\beta }\leq {\left\{\sum_{t=1}^{n}\chi_{t}(\theta_{t}^{ub})\right\}}^{\beta }$ and ${\left\{\sum_{t=1}^{n}\chi_{t}^{\prime }({\left(\zeta \right)}_{t}^{\prime })\right\}}^{\beta }\leq {\left\{\sum_{t=1}^{n}\chi_{t}(\zeta_{t})\right\}}^{\beta }$.
  Therefore, $CIFSSPWA(\varrho_{1},\varrho_{2},...,\varrho_{t})\leq CIFSSPWA(\varrho_{1}^{\prime },\varrho_{2}^{\prime },...,\varrho_{t}^{\prime })$.
**(iii)** $\tau_{min}^{lb}=\min_{t}(\tau_{t}^{lb})$, $\tau_{min}^{ub}=\min_{t}(\tau_{t}^{ub})$, $\theta_{min}^{lb}=\min_{t}(\theta_{t}^{lb})$, $\theta_{min}^{ub}=\min_{t}(\theta_{t}^{ub})$;
  $\tau_{max}^{lb}=\max_{t}(\tau_{t}^{lb})$, $\tau_{max}^{ub}=\max_{t}(\tau_{t}^{ub})$, $\theta_{max}^{lb}=\max_{t}(\theta_{t}^{lb})$, $\theta_{max}^{ub}=\max_{t}(\theta_{t}^{ub})$.
  Assume that
  $CIFSSPWA(\varrho_{1},\varrho_{2},...,\varrho_{n})=\varrho =(<[\tau^{lb},\tau^{ub}],\varepsilon >,<[\theta^{lb},\theta^{ub}],\zeta >)$.
  Then evidently
  (12) $$\left([\tau_{min}^{lb},\tau_{min}^{ub}],[\theta_{max}^{lb},\theta_{max}^{ub}])\leq ([\tau^{lb},\tau^{ub}],[\theta^{lb},\theta^{ub}]\right)$$
  (13) $$([\tau_{max}^{lb},\tau_{max}^{ub}],[\theta_{min}^{lb},\theta_{min}^{ub}])\geq ([\tau^{lb},\tau^{ub}],[\theta^{lb},\theta^{ub}]).$$
  Thus, from (12) and (13), we have
  $\varrho_{min}\leq CIFSSPWA(\varrho_{1},\varrho_{2},...,\varrho_{t})\leq \varrho_{max}$. □

### 3.2 Cubic intuitionistic fuzzy SS power weight geometric aggregation operator

Definition 14 Assume that $\overset{˜}{\varpi }={\left(\varpi_{1},\varpi_{2},...,\varpi_{n}\right)}^{T}$ is weight vector wherein $\varpi_{t}\in [0,1]$ for t∈1,2,...,n such that $\sum_{t=1}^{n}\varpi_{t}=1$. Suppose, there are $\varrho_{t},(t=1,2,...,n)$ CIFNs in this array. Then, a mapping $Z^{n}\times Z^{n}$ → Z×Z is shown as the CIFSSPWG operator, in order that

(14) $$CIFSSPWG(\varrho_{1},\varrho_{2},...,\varrho_{n})=\overset{n}{\underset{t=1}{⨂}}({\left(\varrho_{t}\right)}^{\frac{\varpi_{t}\{1+ℸ(\varrho_{t})\}}{\sum_{t=1}^{n}\varpi_{t}\{1+ℸ(\varrho_{t})\}}}),$$

where $ℸ(\varrho_{t})=\sum_{t=1,t\neq r}^{n}\kappa (\varrho_{t},\varrho r)$ and $\kappa (\varrho_{t},\varrho r)$ indicated support for $\varrho_{t}$ from $\varrho_{r}$.

Theorem 4 *Assume that* $\overset{˜}{\varpi }={\left(\varpi_{1},\varpi_{2},...,\varpi_{n}\right)}^{T}$ *is weight vector wherein* $\varpi_{t}\in [0,1]$ *for* t∈1,2,...,n *such that* $\sum_{t=1}^{n}\varpi_{t}=1$ *. Suppose, there are* $\varrho_{t},(t=1,2,...,n)$ CIFNs *in this array. Then, using the* CIFSSPWG *operator, the aggregate yield remains a CIFN, satisfying*

(15) $$CIFSSPWG(\varrho_{1},\varrho_{2},...,\varrho_{n})=\overset{n}{\underset{t=1}{⨂}}({\left(\varrho_{t}\right)}^{\frac{\varpi_{t}\{1+ℸ(\varrho_{t})\}}{\sum_{t=1}^{n}\varpi_{t}\{1+ℸ(\varrho_{t})\}}})(\langle \left[{\left\{\sum_{t=1}^{n}\chi_{t}{\left(\tau_{t}^{lb}\right)}^{\beta }\right\}}^{1/\beta },{\left\{\sum_{t=1}^{n}\chi_{t}{\left(\tau_{t}^{ub}\right)}^{\beta }\right\}}^{1/\beta }\right],{\left\{\sum_{t=1}^{n}\chi_{t}{\left(\varepsilon_{t}\right)}^{\beta }\right\}}^{1/\beta }\rangle \;,\langle \left[1-{\left\{\sum_{t=1}^{n}\chi_{t}{\left(1-\theta_{t}^{lb}\right)}^{\beta }\right\}}^{1/\beta },1-{\left\{\sum_{t=1}^{n}\chi_{t}{\left(1-\theta_{t}^{ub}\right)}^{\beta }\right\}}^{1/\beta }\right],1-{\left\{\sum_{t=1}^{n}\chi_{t}{\left(1-\zeta_{t}\right)}^{\beta }\right\}}^{1/\beta }\rangle )$$

*where* $\chi_{t}=\frac{\varpi_{t}\{1+ℸ(\varrho_{t})\}}{\sum_{t=1}^{n}\varpi_{t}\{1+ℸ(\varrho_{t})\}},(t=1,2,...,n)$ *and* $ℸ(\varrho_{t})=\sum_{t=1,t\neq r}^{n}\kappa (\varrho_{t},\varrho r)$ *.*

Remark 5 The CIFSSPWG operator also satisfies idempotency, monotonicity, and boundedness properties.

## 4 A technique to handle MCDM problems with cubic intuitionistic fuzzy SS operators

This segment targets to place the CIFSS power aggregation operators at the center of the Multiple Criteria Decision Making (MCDM) method. For each choice, we denote the set of alternatives as K= $\left\{K_{1},K_{2},...,K_{m}\right\}$, and V=$\left\{V_{1},V_{2},...,V_{n}\right\}$ represents the set of attributes using the weight vector $\overset{˜}{\varpi }=\varpi_{1},\varpi_{2},...,\varpi_{n}$ in a way that ${\overset{˜}{\varpi }}_{k}$ ∈ [0,1] and $\sum_{t=1}^{n}{\overset{˜}{\varpi }}_{t}$=1 determine the weight vector $\overset{˜}{\varpi }$, which represents the equivalent significance of different criterion used in the process of making decisions.

**Figure 1:**
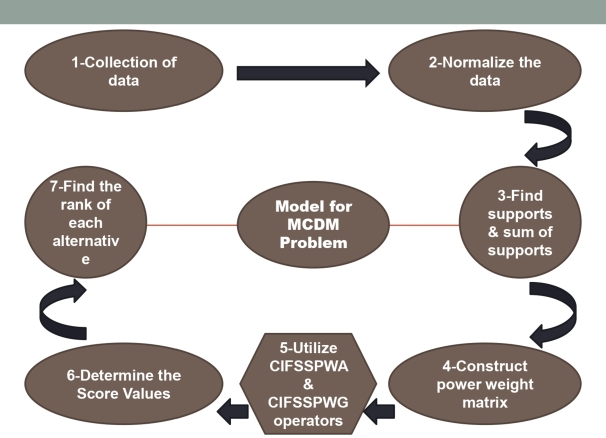
Pictorial Model for MCDM Problem.

Assume that the *CIF* decision matrix is $\overset{˜}{G}={\left(\overset{˜}{g_{kt}}\right)}_{m\times n}=(<[\tau_{kt}^{lb},\tau_{kt}^{ub}],\varepsilon_{kt}>,<[\theta_{kt}^{lb},\theta_{kt}^{ub}],\zeta_{kt}>)$. The degree in which the alternatives $K_{k}$ fulfill the $V_{t}$ characteristic is denoted by $<\tau_{kt},\varepsilon_{kt}>$, and the degree $<\theta_{kt},\zeta >$ to which the alternatives $K_{k}$ do not satisfy the $V_{t}$ traits such that $\tau_{kt},\theta_{kt}\in$[0,1], where the decision-maker provides $\varepsilon_{kt}+\zeta_{kt}\leq 1$ and $\tau_{kt}^{ub}+\theta_{kt}^{ub}\leq 1$. Now, the CIFSSPWA and CIFSSPWG operators are employed to the previously mentioned MCDM troubles in order to evaluate the most appropriate perspectives. To solve MCDM hurdles, the subsequent methodologies are mentioned according to figure [1].

**Step 1.** The following decision-matrix can be used to elaborate the MCDM problem taking into account, that was initially provided within the *CIF* structure:
  (16) $${\overset{˜}{G}}_{m\times n}=\left(\begin{array}{ccccc} {\overset{˜}{g}}_{11} & \ldots & {\overset{˜}{g}}_{1r} & \ldots & {\overset{˜}{g}}_{1n}\\ \vdots & \ddots & \;\vdots & \ddots & \vdots \\ {\overset{˜}{g}}_{i1} & \ldots & {\overset{˜}{g}}_{ir} & \ldots & {\overset{˜}{g}}_{in}\\ \vdots & \ddots & \;\vdots & \ddots & \vdots \\ {\overset{˜}{g}}_{m1} & \ldots & {\overset{˜}{g}}_{mr} & \ldots & {\overset{˜}{g}}_{mn} \end{array}\right)$$
**Step 2.** All at once, many modes of benfit and cost criteria are used to reform the *CIF* assessment matrix ${\overset{˜}{G}}_{m\times n}$. Because each of these two criterion have contradictory actions, the benefit scale works satisfactorily while the cost criterion works worsely when the worth rises.
**Step 3.** For each k∈1,2,...,m, compute the supports of $\varrho_{kt}$ from $\varrho_{kl}(t\neq l)$ to ascertain their proximity to the attribute values of the normalized decision-matrix $\overset{˜}{G}$, (6) being used as
  $$\kappa ({\overset{˜}{\varrho }}_{kt},{\overset{˜}{\varrho }}_{kl})=1-d({\overset{˜}{\varrho }}_{kt},{\overset{˜}{\varrho }}_{kl})$$
  $$=1-\frac{\left(|\tau_{kt}^{lb}-\tau_{kt}^{lb}|+|\tau_{kt}^{ub}-\tau_{kt}^{ub}|+|\varepsilon_{kt}-\varepsilon_{kt}|)+(|\theta_{kt}^{lb}-\theta_{kt}^{lb}|+|\theta_{kt}^{ub}-\theta_{kt}^{ub}|+|\zeta_{kt}+\zeta_{kt}|\right)}{6}$$
  Thus, create the support-matrix
  $$S=[\{S_{tl}^{k};t\neq l,l=1,2,...,n\}],$$
  where $\varrho_{kt}$ from $\varrho_{kl}$ is represented by all supports as a (n-1) tuple vector with the form $S_{tl}^{k}=(\kappa {\left(\varrho_{kt},\varrho_{kl}\right)}_{l=1,l\neq t}^{n}$.
  Next, calculate the sum of supports
  $$ℸ({\overset{˜}{\varrho }}_{kt})=\sum_{l=1,l\neq t}^{n}S_{tl}^{k},$$
  where k∈{1,2,...,m} and t∈{1,2,...,n}.
**Step 4.** With the weights $\varpi_{t}(t=1,2,...,n)$ according to the attribute $S_{t}$ as follows, calculate the weighted proximity of each alternative respecting criteria:
  $$\chi_{kt}=\frac{\varpi_{t}\{1+ℸ(\varrho_{kt}\}}{\sum_{t=1}^{n}\varpi_{t}\{1+ℸ(\varrho_{kt})\}},(t=\{1,2,...,n\})$$
  Consequently, the power weight-matrix is generated.
**Step 5.** Use the CIFSSPWA (or, CIFSSPWG) operator to aggregate all attribute values interlinked with every choice for each β<0. Each alternative will give a single CIFN as a yield.
  (17) $${\overset{˜}{\varrho }}_{k}=CIFSSPWA({\overset{˜}{\varrho }}_{k1},{\overset{˜}{\varrho }}_{k2},...,{\overset{˜}{\varrho }}_{kn})=\frac{{\underset{SS}{⨁}}_{t=1}^{n}(\varpi_{t}\{1+ℸ({\overset{˜}{\varrho }}_{kt})\}{\overset{˜}{\varrho }}_{kt})}{\sum_{t=1}^{n}\varpi_{t}\{1+ℸ({\overset{˜}{\varrho }}_{kt})\}}=(\langle \left[1-{\left\{\sum_{t=1}^{n}\chi_{kt}{\left(1-\tau_{kt}^{lb}\right)}^{\beta }\right\}}^{1/\beta },1-{\left\{\sum_{t=1}^{n}\chi_{kt}{\left(1-\tau_{kt}^{ub}\right)}^{\beta }\right\}}^{1/\beta }\right]\;,1-{\left\{\sum_{t=1}^{n}\chi_{kt}{\left(1-\varepsilon_{kt}\right)}^{\beta }\right\}}^{1/\beta }\rangle \;,\langle \left[{\left\{\sum_{t=1}^{n}\chi_{kt}{\left(\theta_{kt}^{lb}\right)}^{\beta }\right\}}^{1/\beta },{\left\{\sum_{t=1}^{n}\chi_{kt}{\left(\theta_{kt}^{ub}\right)}^{\beta }\right\}}^{1/\beta }\right]\;,{\left\{\sum_{t=1}^{n}\chi_{kt}{\left(\zeta_{kt}\right)}^{\beta }\right\}}^{1/\beta }\rangle )$$
  Or,
  (18) $${\overset{˜}{\varrho }}_{k}=CIFSSPWG({\overset{˜}{\varrho }}_{k1},{\overset{˜}{\varrho }}_{k2},...,{\overset{˜}{\varrho }}_{kn})=\overset{n}{\underset{t=1}{⨂}}({\left({\overset{˜}{\varrho }}_{t}\right)}^{\frac{\varpi_{t}\{1+ℸ({\overset{˜}{\varrho }}_{kt})\}}{\sum_{t=1}^{n}\varpi_{t}\{1+ℸ({\overset{˜}{\varrho }}_{kt})\}}})=(\langle \left[{\left\{\sum_{t=1}^{n}\chi_{kt}{\left(\tau_{kt}^{lb}\right)}^{\beta }\right\}}^{1/\beta },{\left\{\sum_{t=1}^{n}\chi_{kt}{\left(\tau_{kt}^{ub}\right)}^{\beta }\right\}}^{1/\beta }\right]\;,{\left\{\sum_{t=1}^{n}\chi_{kt}{\left(\varepsilon_{kt}\right)}^{\beta }\right\}}^{1/\beta }\rangle \;,\langle \left[1-{\left\{\sum_{t=1}^{n}\chi_{kt}{\left(1-\theta_{kt}^{lb}\right)}^{\beta }\right\}}^{1/\beta },1-{\left\{\sum_{t=1}^{n}\chi_{kt}{\left(1-\theta_{kt}^{ub}\right)}^{\beta }\right\}}^{1/\beta }\right]\;,1-{\left\{\sum_{t=1}^{n}\chi_{kt}{\left(1-\zeta_{kt}\right)}^{\beta }\right\}}^{1/\beta }\rangle )$$
**Step 6.** The Score values of the resulting CIFNs ${\overset{˜}{\varrho }}_{k},k\in 1,2,...,m$ are found from (4). When the score values become equal then we use the accuracy function instead of the score function, which is found from (5).
**Step 7.** In the last stage, the exact-matched signal is picked up after every alternative is ranked on the basis of score values (when equal, then on the basis of accuracy function).

## 5 Numerical example

How our approach to decision-making may improve diabetic care is being demonstrated by us in this phase. Our technique should be flexible enough to treat a wide range of healthcare issues and not just be confined to a limited set of medical ones. Our approach assists researchers, policymakers, and healthcare professionals in making well-informed decisions that lead to the improvement of the patient's outcomes, maximize resource consumption, and ensure the maintenance of diabetes treatment by noticing a variety of parameters and offering an organized decision-making algorithm. Diabetic care experts are significantly invited to examine our approach.

Given the entanglement of diabetes cure, here we recognize key aspects for investigation: Blood Glucose Control $\left(V_{1}\right)$, Patient Adherence and Lifestyle Compatibility $\left(V_{2}\right)$, Risk of Hypoglycemia $\left(V_{3}\right)$, and Cost-Effectiveness $\left(V_{4}\right)$. We then classify different approaches to diabetes management: Insulin Therapy Adjustment $\left(K_{1}\right)$, Oral Medication Optimization $\left(K_{2}\right)$, Lifestyle Modification Program $\left(K_{3}\right)$, and Continuous Glucose Monitoring (CGM) Integration $\left(K_{4}\right)$.

We engage the Cubic Intuitionistic Fuzzy (CIF) framework with the CIFSSPWA and CIFSSPWG operators to label the heterogenous nature of diabetes treatment. This way shows how adaptable it is when dealing with complex medical decision making. To support the advancement of effective and targeted diabetes care by putting patient protection and the long-term continuity of medical techniques is our primary goal.

**Step 1:** Compiling the matrix-formatted data, as shown in Table 1, in order to identify complex relationships and patterns.

**Table 1:** CIF decision-matrix obtained by $\overset{˜}{G}$.

	*V*_1_	*V*_2_	*V*_3_	*V*_4_
*K*_1_	(〈[0.3,0.5],0.3〉,	(〈[0.2,0.5],0.5〉,	(〈[0.1,0.2],0.1〉,	(〈[0.2,0.3],0.2〉,
	〈[0.2,0.4],0.2〉)	〈[0.3,0.4],0.3〉)	〈[0.4,0.7],0.7〉)	〈[0.5,0.6],0.5〉)

*K*_2_	(〈[0.3,0.7],0.4〉,	(〈[0.4,0.5],0.5〉,	(〈[0.1,0.2],0.1〉,	(〈[0.2,0.4],0.2〉,
	〈[0.1,0.2],0.2〉)	〈[0.3,0.4],0.3〉)	〈[0.5,0.7],0.6〉)	〈[0.3,0.4],0.3〉)

*K*_3_	(〈[0.5,0.6],0.6〉,	(〈[0.4,0.5],0.4〉,	(〈[0.1,0.2],0.1〉,	(〈[0.2,0.3],0.2〉,
	〈[0.2,0.3],0.2〉)	〈[0.1,0.2],0.2〉)	〈[0.5,0.7],0.5〉)	〈[0.3,0.4],0.3〉)

*K*_4_	(〈[0.4,0.6],0.4〉,	(〈[0.3,0.7],0.4〉,	(〈[0.1,0.2],0.1〉,	(〈[0.2,0.3],0.2〉,
	〈[0.2,0.3],0.3〉)	〈[0.1,0.2],0.1〉)	〈[0.4,0.6],0.5〉)	〈[0.5,0.6],0.5〉)

**Step 2:** Normalize the data with the assistance of table [2] by applying the above-described technique.

**Table 2:** Matrix Normalization.

	*V*_1_	*V*_2_	*V*_3_	*V*_4_
*K*_1_	(〈[0.3,0.5],0.3〉,	(〈[0.2,0.5],0.5〉,	(〈[0.4,0.7],0.7〉,	(〈[0.5,0.6],0.5〉,
	〈[0.2,0.4],0.2〉)	〈[0.3,0.4],0.3〉)	(〈[0.1,0.2],0.1〉	(〈[0.2,0.3],0.2〉

*K*_2_	(〈[0.3,0.7],0.4〉,	(〈[0.4,0.5],0.5〉,	(〈[0.5,0.7],0.6〉,	(〈[0.3,0.4],0.3〉,
	〈[0.1,0.2],0.2〉)	〈[0.3,0.4],0.3〉)	(〈[0.1,0.2],0.1〉	(〈[0.2,0.4],0.2〉

*K*_3_	(〈[0.5,0.6],0.6〉,	(〈[0.4,0.5],0.4〉,	(〈[0.5,0.7],0.5〉,	(〈[0.3,0.4],0.3〉,
	〈[0.2,0.3],0.2〉)	〈[0.1,0.2],0.2〉)	(〈[0.1,0.2],0.1〉	(〈[0.2,0.3],0.2〉

*K*_4_	(〈[0.4,0.6],0.4〉,	(〈[0.3,0.7],0.4〉,	(〈[0.4,0.6],0.5〉,	(〈[0.5,0.6],0.5〉,
	〈[0.2,0.3],0.3〉)	〈[0.1,0.2],0.1〉)	(〈[0.1,0.2],0.1〉	(〈[0.2,0.3],0.2〉

**Step 3:** For each k={1,2,3,4}, where t,l={1,2,3,4} and t≠r, find the supports of ${\overset{˜}{\varrho }}_{kt}$ from ${\overset{˜}{\varrho }}_{kr}$ by using normalized Hamming distances.

First, let k=1.

Then supports for ${\overset{˜}{\varrho }}_{11}$ from ${\overset{˜}{\varrho }}_{12},{\overset{˜}{\varrho }}_{13},{\overset{˜}{\varrho }}_{14}$ are computed as follows:

$$\kappa ({\overset{˜}{\varrho }}_{11},{\overset{˜}{\varrho }}_{12})=1-d({\overset{˜}{\varrho }}_{11},{\overset{˜}{\varrho }}_{12})$$

$$=1-\frac{\left(|\tau_{kt}^{lb}-\tau_{kt}^{lb}|+|\tau_{kt}^{ub}-\tau_{kt}^{ub}|+|\varepsilon_{kt}-\varepsilon_{kt}|)+(|\theta_{kt}^{lb}-\theta_{kt}^{lb}|+|\theta_{kt}^{ub}-\theta_{kt}^{ub}|+|\zeta_{kt}+\zeta_{kt}|\right)}{6}=0.9167,$$

$\kappa ({\overset{˜}{\varrho }}_{11},{\overset{˜}{\varrho }}_{13})=0.8167,\kappa ({\overset{˜}{\varrho }}_{11},{\overset{˜}{\varrho }}_{14})=0.9000$, respectively.

Therefore, the 3-tupled vector, $S_{1r}^{1}\{r\neq 1,r=2,3,4\}=(0.9167.8167.9000)$ is discovered.

Similarly, the other tuple vectors for k=1 are:

$S_{2r}^{1}(r=1,3,4)=(0.9167.8000.8833)$,

$S_{3r}^{1}(r=1,2,4)=(0.8167.8000.8833)$,

$S_{4r}^{1}(r=1,2,3)=(0.9000.8833.8833)$.

For k=2,3,4 and t=1,2,3,4, the remaining tuples $S_{tr}^{k}(r\neq t,r=1,2,3,4)$ are also obtained in a similar manner.

The support-matrix is therefore as follows:

$$=\left(\begin{array}{cccc} \left(0.9167.8167.9000\right) & \left(0.9167.8000.8833\right) & \left(0.8167.8000.8833\right) & \left(0.9000.8833.8833\right)\\ \left(0.8500.9167.8833\right) & \left(0.8500.8333.9000\right) & \left(0.9167.8333.8000\right) & \left(0.8833.9000.8000\right)\\ \left(0.9000.9167.8833\right) & \left(0.9000.9167.9167\right) & \left(0.9167.9167.8333\right) & \left(0.8833.9167.8333\right)\\ \left(0.9000.9167.9500\right) & \left(0.9000.9500.8833\right) & \left(0.9167.9500.9333\right) & \left(0.9500.8833.9333\right) \end{array}\right)$$

Next, use the support matrix to find the sum of supports $ℸ({\overset{˜}{\varrho }}_{kt})$ for k={1,2,3,4} and t={1,2,3,4}.

The sum of all of the first alternative's supports across various attributes in the given situation of k=1 is as follows:

$ℸ({\overset{˜}{\varrho }}_{11})=\sum_{r=2}^{4}S_{1r}^{1}=2.6334$, and similarly, $ℸ({\overset{˜}{\varrho }}_{12})=2.6000,ℸ({\overset{˜}{\varrho }}_{13})=2.5000,ℸ({\overset{˜}{\varrho }}_{14})=2.6666$.

Presently, the corresponding sum of all supports for k=2,3,4 is found as

$ℸ({\overset{˜}{\varrho }}_{21})=2.6500,ℸ({\overset{˜}{\varrho }}_{22})=2.5833,ℸ({\overset{˜}{\varrho }}_{23})=2.5500,ℸ({\overset{˜}{\varrho }}_{24})=2.5833$;

$ℸ({\overset{˜}{\varrho }}_{31})=2.7000,ℸ({\overset{˜}{\varrho }}_{32})=2.7334,ℸ({\overset{˜}{\varrho }}_{33})=2.6667,ℸ({\overset{˜}{\varrho }}_{34})=2.6333$;

$ℸ({\overset{˜}{\varrho }}_{41})=2.7667,ℸ({\overset{˜}{\varrho }}_{42})=2.7333,ℸ({\overset{˜}{\varrho }}_{43})=2.8000,ℸ({\overset{˜}{\varrho }}_{44})=2.7666$.

**Step 4:** Evaluate the power weights $\chi_{kt}$, in accordance with ${\overset{˜}{\varrho }}_{kt}$ utilizing known weights

{0.4000,0.3000,0.2000,0.1000} are:

$$\chi_{11}=\frac{\varpi_{1}\{1+ℸ(\varrho_{11})\}}{\sum_{t=1}^{4}\varpi_{t}\{1+ℸ(\varrho_{1t})\}}=0.2961$$

In the same way, we compute $\chi_{kt}$ for k=2,3,4 and t=1,2,3,4. At last, the power weight-matrix ${\left(\chi_{kt}\right)}_{4\times 4}$ is obtained.

$$=\left(\begin{array}{cccc} 0.4037 & 0.3000 & 0.1944 & 0.1018\\ 0.4052 & 0.2983 & 0.1970 & 0.0994\\ 0.4004 & 0.3030 & 0.1984 & 0.0983\\ 0.4004 & 0.2976 & 0.2019 & 0.1001 \end{array}\right)$$

**Step 5:** To obtain a single CIFN for each option, aggregate all attribute values corresponding to each alternative using the CIFSSPWA operator (and, CIFSSPWG operator) for β=−1.

$${\overset{˜}{\varrho }}_{1}=CIFSSPWA({\overset{˜}{\varrho }}_{11},{\overset{˜}{\varrho }}_{12},{\overset{˜}{\varrho }}_{13},{\overset{˜}{\varrho }}_{14})=(\langle \left[1-{\left\{\sum_{t=1}^{4}\chi_{1t}{\left(1-\tau_{1t}^{lb}\right)}^{\beta }\right\}}^{1/\beta },1-{\left\{\sum_{t=1}^{4}\chi_{1t}{\left(1-\tau_{1t}^{ub}\right)}^{\beta }\right\}}^{1/\beta }\right]\;,1-{\left\{\sum_{t=1}^{4}\chi_{1t}{\left(1-\varepsilon_{1t}\right)}^{\beta }\right\}}^{1/\beta }\rangle \;,\langle \left[{\left\{\sum_{t=1}^{4}\chi_{1t}{\left(\theta_{1t}^{lb}\right)}^{\beta }\right\}}^{1/\beta },{\left\{\sum_{t=1}^{4}\chi_{1t}{\left(\theta_{1t}^{ub}\right)}^{\beta }\right\}}^{1/\beta }\right]\;,{\left\{\sum_{t=1}^{4}\chi_{1t}{\left(\zeta_{1t}\right)}^{\beta }\right\}}^{1/\beta }\rangle )=\left(\langle \left[0.3240,0.5671\right],0.5070\rangle ,\langle \left[0.1828,0.3257\right],0.1828\rangle \right).$$

Similarly, ${\overset{˜}{\varrho }}_{2}=\left(\langle \left[0.3797,0.6389\right],0.4755\rangle ,\langle \left[0.1331,0.2497\right],0.1822\rangle \right)$,

${\overset{˜}{\varrho }}_{3}=\left(\langle \left[0.4574,0.5888\right],0.5106\rangle ,\langle \left[0.1332,0.2398\right],0.1669\rangle \right)$,

${\overset{˜}{\varrho }}_{4}=\left(\langle \left[0.3862,0.6361\right],0.4342\rangle ,\langle \left[0.1334,0.2400\right],0.1464\rangle \right)$.

and

$${\overset{˜}{\varrho }}_{1}=CIFSSPWG({\overset{˜}{\varrho }}_{11},{\overset{˜}{\varrho }}_{12},{\overset{˜}{\varrho }}_{13},{\overset{˜}{\varrho }}_{14})=(\langle \left[{\left\{\sum_{t=1}^{4}\chi_{1t}{\left(\tau_{1t}^{lb}\right)}^{\beta }\right\}}^{1/\beta },{\left\{\sum_{t=1}^{4}\chi_{1t}{\left(\tau_{1t}^{ub}\right)}^{\beta }\right\}}^{1/\beta }\right]\;,{\left\{\sum_{t=1}^{4}\chi_{1t}{\left(\varepsilon_{1t}\right)}^{\beta }\right\}}^{1/\beta }\rangle \;,\langle \left[1-{\left\{\sum_{t=1}^{4}\chi_{1t}{\left(1-\theta_{1t}^{lb}\right)}^{\beta }\right\}}^{1/\beta },1-{\left\{\sum_{t=1}^{4}\chi_{1t}{\left(1-\theta_{1t}^{ub}\right)}^{\beta }\right\}}^{1/\beta }\right]\;,1-{\left\{\sum_{t=1}^{4}\chi_{1t}{\left(1-\zeta_{1t}\right)}^{\beta }\right\}}^{1/\beta }\rangle )=\left(\langle \left[0.2829,0.5391\right],0.4120\rangle ,\langle \left[0.2166,0.3595\right],0.2166\rangle \right).$$

Similarly, ${\overset{˜}{\varrho }}_{2}=\left(\langle \left[0.3544,0.5864\right],0.4407\rangle ,\langle \left[0.1800,0.2936\right],0.2162\rangle \right)$,

${\overset{˜}{\varrho }}_{3}=\left(\langle \left[0.4381,0.5548\right],0.4653\rangle ,\langle \left[0.1529,0.2533\right],0.1821\rangle \right)$,

${\overset{˜}{\varrho }}_{4}=\left(\langle \left[0.3707,0.6266\right],0.4257\rangle ,\langle \left[0.1530,0.2534\right],0.2014\rangle \right)$.

**Step 6:** Using CIFSSPWA operator, the Score values of the resulting CIFNs ${\overset{˜}{\varrho }}_{k},k=\{1,2,3,4\}$ are derived from (4) as $S(\varrho_{1})=0.4527,S(\varrho_{2})=0.4762,S(\varrho_{3})=0.4988,S(\varrho_{4})=0.4722$.

and using CIFSSPWG operator, the Score values of the resulting CIFNs ${\overset{˜}{\varrho }}_{k},k=\{1,2,3,4\}$ are calculated as $S(\varrho_{1})=0.3868,S(\varrho_{2})=0.4263,S(\varrho_{3})=0.4588,S(\varrho_{4})=0.4407$.

**Step 7:** After ranking the options according to score values, the following is the outcome for the CIFSSPWA operator:

$$K_{3}>K_{2}>K_{4}>K_{1},$$

and the following is the outcome for the CIFSSPWG operator:

$$K_{3}>K_{4}>K_{2}>K_{1}.$$

$K_{3}$ is decided to be the quality choice since $K_{3}$ has the highest possible score value from all of the choices for both operators.

## 6 Sensitivity analysis

Sensitivity analysis is a kind of financial model that is used to observe what changes in input elements impose an impact on the track factors. Predictions made in this way rely on key variables, including the sensitivity of a parameter (denoted as β<0) to certain weights. The two sequences generated by the CIFSSPWA and CIFSSPWG operators have somehow different substitutes, but $K_{3}$ is still the best selection. Sensitivity analysis is important as it controls uncertainty in mathematical models like variations in the input values. For the enhancement of the accuracy of analysis and models that depend on assumptions regarding input precision, it is frequently employed in conjunction with uncertainty examination. Applying sensitivity analysis aids with computation, forecasting, and finding fields that need improvements or adjustments in cycles. It is important to notice that exclusive dependence on historical data might result in forecasts that are not correct because past events do not always indicate future ones.

### 6.1 Sensitivity for “*β*” parameter

**Figure 2:**
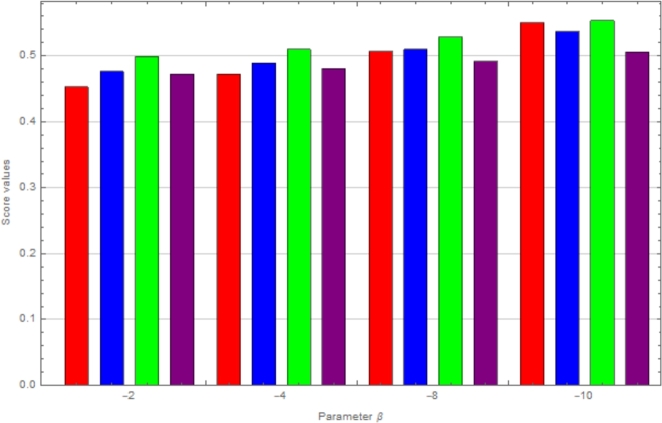
Graphical representation by using CIFSSPWA operator.

**Figure 3:**
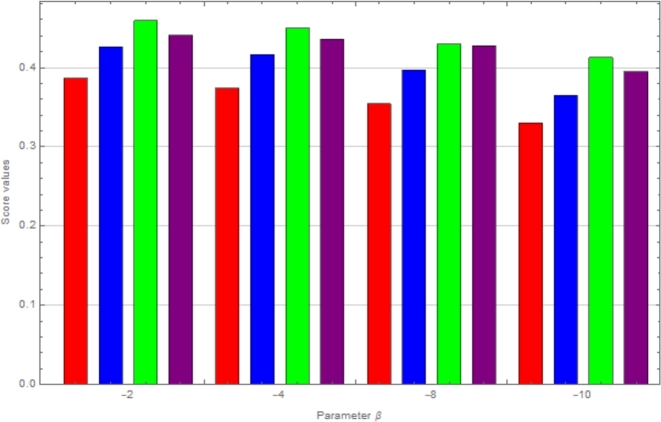
Graphical representation by using CIFSSPWG operator.

In this passage, we observe sensitivity using *β*-CIFSSPWA and *β*-CIFSSPWG operators from tables [3] and [4]. We analyze how varying β<0 affects the ranking of choices. The Figure 2, Figure 3 show that reducing *β* hardly varies results. As *β* decreases, option scores reduce yet the top choice, $K_{3}$, remains constant.

**Table 3:** Using the CIFSSPWA operator to change the parameter values for ranking.

*β*-values:	Score Values	Ranking
*β*=-1	$S(\varrho_{1})=0.4527$, $S(\varrho_{2})=0.4762$, $S(\varrho_{3})=0.4988$, $S(\varrho_{4})=0.4722$	*K*_3_ > *K*_2_ > *K*_4_ > *K*_1_
*β*=-2	$S(\varrho_{1})=0.4724$, $S(\varrho_{2})=0.4891$, $S(\varrho_{3})=0.5097$, $S(\varrho_{4})=0.4804$	*K*_3_ > *K*_2_ > *K*_4_ > *K*_1_
*β*=-4	$S(\varrho_{1})=0.5068$, $S(\varrho_{2})=0.5100$, $S(\varrho_{3})=0.5288$, $S(\varrho_{4})=0.4916$	*K*_3_ > *K*_2_ > *K*_1_ > *K*_4_
*β*=-8	$S(\varrho_{1})=0.5502$, $S(\varrho_{2})=0.5371$, $S(\varrho_{3})=0.5535$, $S(\varrho_{4})=0.5057$	*K*_3_ > *K*_1_ > *K*_2_ > *K*_4_

**Table 4:** Using the CIFSSPWG operator to change the parameter values for ranking.

*β*-values:	Score Values	Ranking
*β*=-1	$S(\varrho_{1})=0.3868$, $S(\varrho_{2})=0.4263$, $S(\varrho_{3})=0.4588$, $S(\varrho_{4})=0.4407$	*K*_3_ > *K*_4_ > *K*_2_ > *K*_1_
*β*=-2	$S(\varrho_{1})=0.3742$, $S(\varrho_{2})=0.4162$, $S(\varrho_{3})=0.4493$, $S(\varrho_{4})=0.4361$	*K*_3_ > *K*_4_ > *K*_2_ > *K*_1_
*β*=-4	$S(\varrho_{1})=0.3545$, $S(\varrho_{2})=0.3968$, $S(\varrho_{3})=0.4297$, $S(\varrho_{4})=0.4274$	*K*_3_ > *K*_4_ > *K*_2_ > *K*_1_
*β*=-8	$S(\varrho_{1})=0.3301$, $S(\varrho_{2})=0.3648$, $S(\varrho_{3})=0.4127$, $S(\varrho_{4})=0.3949$	*K*_3_ > *K*_4_ > *K*_2_ > *K*_1_

### 6.2 Performing sensitivity analysis with respect to attribute weighting

**Figure 4:**
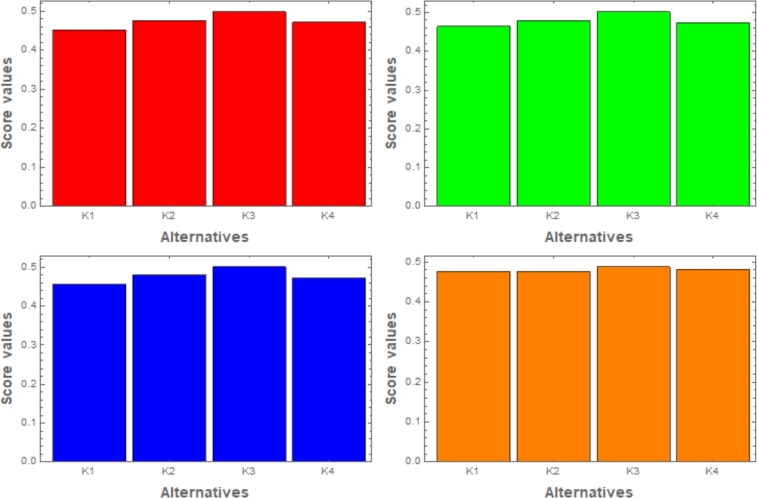
Using CIFSSPWA operator, the relation between weights and ranking.

**Figure 5:**
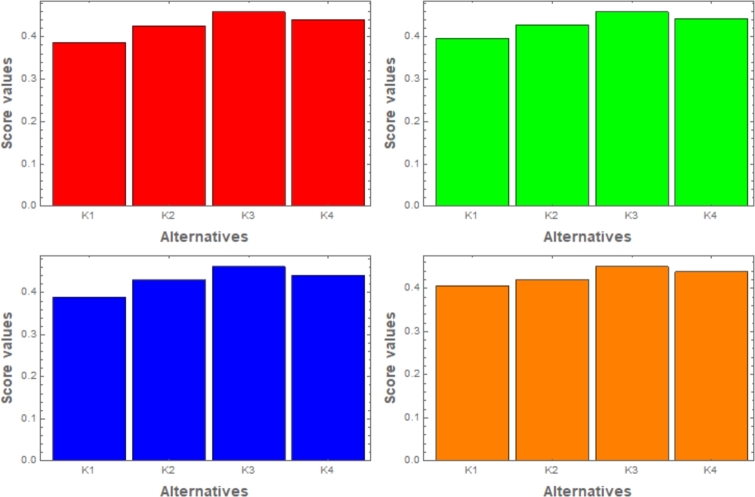
Using CIFSSPWG operator, the relation between weights and ranking.

Sensitivity examination on attribute weights is a rapid technique used in decision-making, most importantly in MCDM. It manipulates the effects of varying weights assigned to evaluated qualities. Attributes are mostly given different weights on the basis of preferences, expertise, or other parameters. The analysis involves adjusting weights and observing how it affects the decision's final stage. Identifying influential attributes and understanding how decisions are affected by altering weights is our main objective. This study helps healthcare professionals, researchers, and policymakers in improving patient results and resource usage in curing diabetes. In short, for examining decision-making problems and approaching their adaptability to changes in attribute weights, sensitivity analysis is very crucial (see in tables [5] and [6]). The figures [4] and [5] depict that the alternate weight assignment leads to a little shift in the classification of alternatives, with $K_{3}$ being the best choice.

**Table 5:** Sensitivity analysis employing the CIFSSPWA operator with respect to weights.

Weights:	Score Values	Ranking
{0.4000,0.3000,0.2000,0.1000}	$S(\varrho_{1})=0.4527$, $S(\varrho_{2})=0.4762$, $S(\varrho_{3})=0.4988$, $S(\varrho_{4})=0.4722$	*K*_3_ > *K*_2_ > *K*_4_ > *K*_1_
{0.4000,0.2500,0.2250,0.1250}	$S(\varrho_{1})=0.4641$, $S(\varrho_{2})=0.4795$, $S(\varrho_{3})=0.5019$, $S(\varrho_{4})=0.4738$	*K*_3_ > *K*_2_ > *K*_4_ > *K*_1_
{0.3940,0.2963,0.2173,0.0924}	$S(\varrho_{1})=0.4568$, $S(\varrho_{2})=0.4802$, $S(\varrho_{3})=0.5016$, $S(\varrho_{4})=0.4728$	*K*_3_ > *K*_2_ > *K*_4_ > *K*_1_
{0.3096,0.2586,0.2345,0.1973}	$S(\varrho_{1})=0.4759$, $S(\varrho_{2})=0.4762$, $S(\varrho_{3})=0.4874$, $S(\varrho_{4})=0.4813$	*K*_3_ > *K*_4_ > *K*_2_ > *K*_1_

**Table 6:** Sensitivity analysis employing the CIFSSPWG operator with respect to weights.

Weights:	Score Values	Ranking
{0.4000,0.3000,0.2000,0.1000}	$S(\varrho_{1})=0.3868$, $S(\varrho_{2})=0.4263$, $S(\varrho_{3})=0.4588$, $S(\varrho_{4})=0.4407$	*K*_3_ > *K*_4_ > *K*_2_ > *K*_1_
{0.4000,0.2500,0.2250,0.1250}	$S(\varrho_{1})=0.3964$, $S(\varrho_{2})=0.4281$, $S(\varrho_{3})=0.4589$, $S(\varrho_{4})=0.4426$	*K*_3_ > *K*_4_ > *K*_2_ > *K*_1_
{0.3940,0.2963,0.2173,0.0924}	$S(\varrho_{1})=0.3892$, $S(\varrho_{2})=0.4299$, $S(\varrho_{3})=0.4621$, $S(\varrho_{4})=0.4414$	*K*_3_ > *K*_4_ > *K*_2_ > *K*_1_
{0.3096,0.2586,0.2345,0.1973}	$S(\varrho_{1})=0.4066$, $S(\varrho_{2})=0.4195$, $S(\varrho_{3})=0.4507$, $℘({\overset{˜}{\varrho }}_{4})=0.4383$	*K*_3_ > *K*_4_ > *K*_2_ > *K*_1_

## 7 Comparison analysis

We conduct the following comparability resemblance to demonstrate the strengths and benefits of the proposed techniques. When we compare our recommended operators with other available ones, we notice that our operators are much more flexible than others. We get optimal alternatives like the ones in table [7] using parameter exactly equal to 2 when compared with Intuitionistic cubic fuzzy Hamacher weighted average(ICFHWA) operator suggested by Muneeza etal. [35]. Our operators also generate the same optimal substitutes as those in the table [7], when matched with CIFOWA and CIFOWG operators that are presented by Muneeza and Abdullah [36]. Here the optimal choice is the same but our proposed operators provide more accuracy through the parameter *β* while the operators provided by Muneeza and Abdullah give less stability and reliance on the outcomes because of no parameter involved.

**Table 7:** Comparative Approaches.

Approaches	Score Values	Ranking
ICFHWA operator [35]	$S(\varrho_{1})=0.4298$, $S(\varrho_{2})=0.4590$, $S(\varrho_{3})=0.4850$, $S(\varrho_{4})=0.4601$	*K*_3_ > *K*_4_ > *K*_2_ > *K*_1_
CIFOWA operator [36]	$S(\varrho_{1})=0.6544$, $S(\varrho_{2})=0.6607$, $S(\varrho_{3})=0.6732$, $S(\varrho_{4})=0.6681$	*K*_3_ > *K*_4_ > *K*_2_ > *K*_1_
CIFOWG operator [36]	$S(\varrho_{1})=0.1746$, $S(\varrho_{2})=0.1883$, $S(\varrho_{3})=0.2009$, $S(\varrho_{4})=0.2006$	*K*_3_ > *K*_4_ > *K*_2_ > *K*_1_
CIFWA operator (when *q*(*t*)=−*log*(*t*)) [37]	$S(\varrho_{1})=0.4780$, $S(\varrho_{2})=0.5000$, $S(\varrho_{3})=0.5249$, $S(\varrho_{4})=0.4976$	*K*_3_ > *K*_2_ > *K*_4_ > *K*_1_
CIFSSPWA (proposed operator)	$S(\varrho_{1})=0.4527$, $S(\varrho_{2})=0.4762$, $S(\varrho_{3})=0.4988$, $S(\varrho_{4})=0.4722$	*K*_3_ > *K*_2_ > *K*_4_ > *K*_1_
CIFSSPWG (proposed operator)	$S(\varrho_{1})=0.3868$, $S(\varrho_{2})=0.4263$, $S(\varrho_{3})=0.4588$, $S(\varrho_{4})=0.4407$	*K*_3_ > *K*_4_ > *K*_2_ > *K*_1_

The optimal substitutes that take place when we match our average operator with the CIFWA operator suggested by Kaur and Garg [37] are shown in table [7]. Despite similar surroundings, our SS operations-based aggregation operators provide simplicity and more accuracy by using the parameter β<0. As CIFS stands as the most progressive framework, the existing fuzzy aggregation methods often try to cope with the complexity of the consisting data. This points to the confined scope of the present aggregation methodologies. So, in comparison with other measures at present, our suggested technique is best fitted to solve decision-making problems.

In table [8], the benefits of our proposed operators are demonstrated. The CIFSSPWA and CIFSSPWG operators are developed by using SS operations together with power aggregation processes, involving parameter *β*. These techniques possess apparent limitations while becoming unusual, especially when implemented in dynamic fuzzy situations. Their usage in practical circumstances associated with large data processing is restricted by their propensity to diminish performance when interacting with massive data sets. Furthermore, the step of choosing appropriate parameters for these operators is tedious and usually entails experimenting, adding a subjective component that could restrict their applicability in everyday situations. Also, whereas such operators enable an advanced structure for making choices regardless of ambiguity, it is essential to thoughtfully evaluate their efficacy in various kinds of circumstances as they might not precisely match the choices of the various decision-makers.

**Table 8:** Comparing the distinctive features of different approaches.

Approaches	Whether express fuzzy data simpler	Whether based on norms	Whether to add the parameter to improve the adaptability of the aggregations
ICFHWA operator [35]	✓	×	✓
CIFOWA operator [36]	✓	×	×
CIFOWG operator [36]	✓	×	×
CIFWA operator (when *q*(*t*)=−*log*(*t*)) [37]	✓	✓	×
CIFSSPWA (proposed operator)	✓	✓	✓
CIFSSPWG (proposed operator)	✓	✓	✓

## 8 Conclusions

The main contribution of this research is the initiation of several effective CIF aggregate operators, especially the CIFSSPWA and CIFSSPWG operators. These operators increase information fusion flexibility when assembled with SS operations. SS operations are essential in aggregation theory because they offer adaptable, expressive, and reliable techniques for integrating different information within unified decision-making processes. When contrasting these operators with previous ones, our proposed operators provide the same selection more simply and give more reliability for complex data. Their efficiency in making accurate judgments by compensating for the effect of wrong input from decision-makers is an important aspect. By utilizing these operators, a new method for managing MCDM with CIF information is developed.

Our productive use of Cubic Intuitionistic Fuzzy SS Power Aggregation Operators in diabetes care entitles healthcare providers to make familiar decisions that cause a big improvement in patient outcomes and allocate the resources effectively, ensuring the long-term continuity of diabetes treatment. Moreover, we highlight the effect of the SS component on grading outcomes by focusing on the benefits of our technique. We express both, the effectiveness and sensibility of our proposed operators in decision-making scenarios through contrasting analyses.

Stepping ahead, there is potential to extend these operators to less clear-cut areas like quasirung fuzzy sets [38], p,q-cubic quasi-rung fuzzy, cubic hesitant fuzzy [39], q-rung orthopair fuzzy N-soft sets [40], interval-valued q-rung orthopair fuzzy soft fuzzy [41] cubic Intuitionistic dual hesitant CIDH, q-rung CI fuzzy, q-rung CIP fuzzy, and others. We hope our proposed analysis will lead to new methodologies for addressing decision-making challenges across different fuzzy contexts.

## CRediT authorship contribution statement

**Bai Chunsong:** Resources, Funding acquisition. **Usman Khalid:** Data curation. **Muhammad Ahsan Binyamin:** Visualization, Validation, Software, Methodology. **Jawad Ali:** Project administration.

## Declaration of Competing Interest

The authors declare that they have no known competing financial interests or personal relationships that could have appeared to influence the work reported in this paper.

## Data Availability

No data was used for the research described in the article.

## References

[1] Zadeh L.A. (1965). Fuzzy sets. Inf. Control.

[2] Chen S.-M., Tanuwijaya K. (2011). Fuzzy forecasting based on high-order fuzzy logical relationships and automatic clustering techniques. Expert Syst. Appl..

[3] Chen S.-M., Munif A., Chen G.-S., Liu H.-C., Kuo B.-C. (2012). Fuzzy risk analysis based on ranking generalized fuzzy numbers with different left heights and right heights. Expert Syst. Appl..

[4] Garg H. (2018). Some arithmetic operations on the generalized sigmoidal fuzzy numbers and its application. Granul. Comput..

[5] Garg H., Ansha (2018). Arithmetic operations on generalized parabolic fuzzy numbers and its application. Proc. Natl. Acad. Sci. India Sect. A Phys. Sci..

[6] Wang H.-Y., Chen S.-M. (2008). Evaluating students' answerscripts using fuzzy numbers associated with degrees of confidence. IEEE Trans. Fuzzy Syst..

[7] Atanassov K.T., Stoeva S. (1986). Intuitionistic fuzzy sets. Fuzzy Sets Syst..

[8] Atanassov K.T., Atanassov K.T. (1999). Intuitionistic Fuzzy Sets: Theory and Applications.

[9] Garg H. (2016). Generalized intuitionistic fuzzy interactive geometric interaction operators using Einstein t-norm and t-conorm and their application to decision making. Comput. Ind. Eng..

[10] Garg H. (2017). Novel intuitionistic fuzzy decision making method based on an improved operation laws and its application. Eng. Appl. Artif. Intell..

[11] Liu X., Wang L. (2020). An extension approach of topsis method with owad operator for multiple criteria decision-making. Granul. Comput..

[12] Mahmood T., Liu P., Ye J., Khan Q. (2018). Several hybrid aggregation operators for triangular intuitionistic fuzzy set and their application in multi-criteria decision making. Granul. Comput..

[13] Liu P., Chen S.-M. (2016). Group decision making based on Heronian aggregation operators of intuitionistic fuzzy numbers. IEEE Trans. Cybern..

[14] Wang X. (2008). Fuzzy number intuitionistic fuzzy arithmetic aggregation operators. Int. J. Fuzzy Syst..

[15] Liu P. (2013). Some Hamacher aggregation operators based on the interval-valued intuitionistic fuzzy numbers and their application to group decision making. IEEE Trans. Fuzzy Syst..

[16] Seikh M.R., Mandal U. (2021). Intuitionistic fuzzy Dombi aggregation operators and their application to multiple attribute decision-making. Granul. Comput..

[17] Mandal U., Seikh M.R. (2023). Interval-valued spherical fuzzy mabac method based on Dombi aggregation operators with unknown attribute weights to select plastic waste management process. Appl. Soft Comput..

[18] Jun Y.B., Kim C.S., Yang K.O. (2012). Cubic sets. Ann. Fuzzy Math. Inform..

[19] Mahmood T., Mehmood F., Khan Q. (2016). Cubic hesitant fuzzy sets and their applications to multi criteria decision making. Int. J. Algebra Stat..

[20] Khan M., Abdullah S., Zeb A., Majid A. (2016). Cucbic aggregation operators. Int. J. Comput. Sci. Inf. Secur..

[21] Kaur G., Garg H. (2018). Multi-attribute decision-making based on Bonferroni mean operators under cubic intuitionistic fuzzy set environment. Entropy.

[22] Seikh M.R., Mandal U. (2023). q-rung orthopair fuzzy Archimedean aggregation operators: application in the site selection for software operating units. Symmetry.

[23] Peng X., Yang Y. (2016). Fundamental properties of interval-valued Pythagorean fuzzy aggregation operators. Int. J. Intell. Syst..

[24] Schweizer B., Sklar A. (2011).

[25] Zhang X., Xu Z. (2014). Extension of topsis to multiple criteria decision making with Pythagorean fuzzy sets. Int. J. Intell. Syst..

[26] Wang P., Liu P. (2019). Some Maclaurin symmetric mean aggregation operators based on Schweizer-Sklar operations for intuitionistic fuzzy numbers and their application to decision making. J. Intell. Fuzzy Syst..

[27] Zhang H., Wang F., Geng Y. (2019). Multi-criteria decision-making method based on single-valued neutrosophic Schweizer–Sklar muirhead mean aggregation operators. Symmetry.

[28] Gohain B., Chutia R., Dutta P. (2023). A distance measure for optimistic viewpoint of the information in interval-valued intuitionistic fuzzy sets and its applications. Eng. Appl. Artif. Intell..

[29] Muneeza, Abdullah S., Qiyas M., Khan M.A. (2021). Multi-criteria decision making based on intuitionistic cubic fuzzy numbers. Granul. Comput..

[30] Garg H., Kaur G. (2020). Novel distance measures for cubic intuitionistic fuzzy sets and their applications to pattern recognitions and medical diagnosis. Granul. Comput..

[31] Biswas A., Deb N. (2021). Pythagorean fuzzy Schweizer and Sklar power aggregation operators for solving multi-attribute decision-making problems. Granul. Comput..

[32] Yager R.R. (2001). The power average operator. IEEE Trans. Syst. Man Cybern., Part A, Syst. Hum..

[33] Xu Z., Yager R.R. (2009). Power-geometric operators and their use in group decision making. IEEE Trans. Fuzzy Syst..

[34] Luo M.-X., Cheng Z. (2016). Robustness of fuzzy reasoning based on Schweizer–Sklar interval-valued t-norms. Fuzzy Inf. Eng..

[35] Muneeza, Abdullah S., Aslam M. (2020). New multicriteria group decision support systems for small hydropower plant locations selection based on intuitionistic cubic fuzzy aggregation information. Int. J. Intell. Syst..

[36] Muneeza, Abdullah S. (2020). Multicriteria group decision-making for supplier selection based on intuitionistic cubic fuzzy aggregation operators. Int. J. Fuzzy Syst..

[37] Kaur G., Garg H. (2019). Generalized cubic intuitionistic fuzzy aggregation operators using t-norm operations and their applications to group decision-making process. Arab. J. Sci. Eng..

[38] Seikh M.R., Mandal U. (2022). Multiple attribute group decision making based on quasirung orthopair fuzzy sets: application to electric vehicle charging station site selection problem. Eng. Appl. Artif. Intell..

[39] Mehmood F., Hayat K., Mahmood T., Cao B.-Y. (2019). A multi criteria decision making method for cubic hesitant fuzzy sets based on Einstein operational laws. Ital. J. Pure Appl. Math..

[40] Raja M.S., Hayat K., Munshi A., Mahmood T., Sheraz R., Matloob I. (2024). Aggregation operators on group-based generalized q-rung orthopair fuzzy n-soft sets and applications in solar panel evaluation. Heliyon.

[41] Yang X., Hayat K., Raja M.S., Yaqoob N., Jana C. (2022). Aggregation and interaction aggregation soft operators on interval-valued q-rung orthopair fuzzy soft environment and application in automation company evaluation. IEEE Access.

